# Boron‐10 carriers and their applications in boron neutron capture therapy

**DOI:** 10.1002/pro6.70061

**Published:** 2026-04-08

**Authors:** Dachao Tang, Mengqi Shi, Zhichen Mao, Weiya Chen, Xiaoling Li, Xiao Yan Zhao, Zhigang Liu, Xiao Xu

**Affiliations:** ^1^ School of Petrochemical Engineering Changzhou University Changzhou China; ^2^ Guangdong Engineering Research Center of Boron Neutron Therapy and Application in Malignant Tumors The Tenth Affiliated Hospital Southern Medical University (Dongguan People's Hospital) Southern Medical University Dongguan China; ^3^ Departments of Diagnostic Radiology Yong Loo Lin School of Medicine National University of Singapore Singapore Singapore; ^4^ Cancer Center Dongguan Engineering Research Center for Innovative Boron Drugs and Novel Radioimmune Drugs The Tenth Affiliated Hospital Southern Medical University (Dongguan People's Hospital) Southern Medical University Dongguan China; ^5^ School of Pharmacy China Pharmaceutical University Nanjing Jiangsu China

**Keywords:** Boron neutron capture therapy, boron‐10 carrier, high tumor affinity, L‐p‐boronophenylalanine, sodium borocaptate

## Abstract

Boron neutron capture therapy (BNCT) has emerged as a promising therapeutic modality in cancer treatment, demonstrating the ability to selectively eliminate cancer cells through the ^10^B(n,α)^7^Li nuclear reaction with minimal side effects on normal tissues. As a binary, target‐specific therapeutic modality for malignancies, BNCT critically depends on novel boron delivery carriers that exhibit high tumor affinity and prolonged intratumoral retention. Although several boron carriers have received Food and Drug Administration approval for clinical investigation, leading carriers such as L‐p‐boronophenylalanine (BPA) and sodium borocaptate (BSH) continue to require high infusion doses and exhibit limited tumor selectivity and affinity, short systemic half‐lives, and limited in vivo stability. These challenges have stimulated extensive global research into novel boron‐10 carriers and innovative carrier platforms, including amino acids, sugars, porphyrin derivatives, nucleotides, and a variety of nanocarrier systems. This review provides a systematic classification of high‐abundance boron‐10 carriers, critically examines radio‐boron research, and evaluates the potential integration of BNCT into clinical diagnostics and advanced cancer treatment protocols. By detailing the current status of novel boron‐10 carriers, this review further aims to elucidate critical challenges and opportunities in BNCT drug development, ultimately providing a theoretical foundation for next‐generation BNCT interventions.

## Graphical abstract

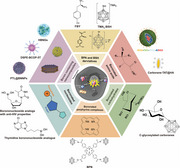

## 1 INTRODUCTION

Boron neutron capture therapy (BNCT)—a binary, targeted, cellular‐level precision radiotherapy approach—integrates principles from nuclear physics, chemistry, biology, medicine, and other disciplines.^1^, ^2^, ^3^ The conceptual foundation of BNCT was laid by the seminal discovery of neutrons by Chadwick in 1932,^4^ followed by Locher's recognition of the therapeutic potential of BNCT in 1936.^5^ The initial clinical implementation of BNCT began in the 1950s when Massachusetts General Hospital (MGH) neurosurgeon William Herbert Sweet conducted pioneering treatments for patients with malignant glioblastoma using the Brookhaven Graphite Research Reactor and MIT Nuclear Research Reactor (MITR).^6^ While BNCT clinical application gradually declined in the United States, this therapeutic approach continued to evolve globally, particularly in Japan, the Netherlands, Finland, Italy, Sweden, and Argentina. A pivotal figure in this global expansion was Japanese physician Hiroshi Hatanaka who, following his training at MGH, spearheaded BNCT research in Japan. Hatanaka's pioneering work focused on treating malignant brain tumors using sodium borocaptate (BSH). Between 1968 and 1993, he treated approximately 120 patients with high‐grade gliomas and achieved promising outcomes.^7^, ^8^, ^9^ The introduction of L‐p‐boronophenylalanine (BPA) as a therapeutic agent in 1987 marked another significant milestone in Japan, particularly in melanoma treatment.^10^ The forefront position of Japan in BNCT development continued with several other achievements: the first case of ^18^F‐BPA‐Positron Emission Tomography (PET) imaging in 1991,^11^ successful visualization of brain tumors using ^18^F‐BPA‐PET tracer in 1994, and Professor Ono's breakthrough at Kyoto University in 2002, demonstrating effective malignant glioma suppression through combined BPA‐ and BSH‐based therapy.^12^, ^13^ BNCT research in Japan continued to progress through the development of accelerator‐based BNCT (AB‐BNCT). The Kansai BNCT Medical Center employs the NeuCure® system developed by Sumitomo Heavy Industries (utilizing a 30 MeV proton beam to bombard a beryllium target), which has treated over 300 patients with head and neck cancers since its inclusion in the national health insurance system in Japan in 2020. In 2025, the Niiyamate Hospital of the Japan Anti‐Tuberculosis Association conducted preliminary clinical research at the Kyoto University Research Reactor, using intravenous injection of BPA for BNCT as part of an intensified combination therapy for locally recurrent and metastatic cancers in the gastrointestinal region.^14^

The 1990s marked a significant expansion of BNCT across Europe. The Netherlands employed the High Flux Reactor for glioblastoma treatment,^15^ while Sweden conducted BNCT treatments for patients with brain tumors at the Studsvik reactor facility between 2001 and 2002. Finland emerged as a particularly successful center, utilizing the FiR‐1 reactor to establish a comprehensive clinical program focusing on head and neck cancer and recurrent brain tumors. This program achieved notable success, with the second‐highest patient treatment volume globally after Japan.^16^, ^17^ Taiwan has also made remarkable contributions to BNCT research and clinical application. Since 1992, a collaborative research team from National Tsing Hua University (NTHU) and Taipei Veterans General Hospital has conducted extensive BNCT research, culminating in the completion of a high‐quality epithermal neutron beam BNCT renovation project in 2004. A milestone trilateral academic exchange agreement was established in 2010 between the BNCT facility at NTHU, Taipei Veterans General Hospital, and Kyoto University, paving the way for clinical trials targeting recurrent head and neck cancers. The first phase of clinical trials (2010 – January 2014) completed 32 irradiation sessions for 17 patients with recurrent head and neck cancers. The second phase commenced immediately afterward and expanded in 2016 to include emergency (compassionate) medical treatments. This expansion proved highly successful, with nearly 60 patients receiving treatment, including international cases from Singapore, Japan, Australia, and Mainland China. In 2020, the world's first AB‐BNCT clinical irradiation system was successfully constructed in Japan and officially put into clinical use. Subsequently, countries such as the United States, Russia, the United Kingdom, Italy, Israel, and Argentina have also completed the construction of AB‐BNCT systems.^18^

China has made remarkable progress in BNCT research in recent years, demonstrating therapeutic potential in clinically challenging malignancies, including melanoma, gliomas, and recurrent head and neck cancers.^19^ Early clinical studies at the Taipei Tsinghua Nuclear Reactor in the Taiwan Province provided foundational evidence for the safety and feasibility of BNCT in recurrent head and neck cancer and high‐grade gliomas. Building on these results, accelerator‐based BNCT platforms developed by Heyuan Technology have advanced to phase I clinical trials. In China, several clinical programs have progressed into registered studies. Two prominent initiatives, the IHNI‐1 and NeuPEX platforms, have entered clinical research stages. The IHNI‐1 system has shown encouraging preliminary results in malignant melanoma, with formal outcomes pending further observation and evaluation. In parallel, a phase I clinical trial at Dongguan People's Hospital, which hosts China's first hospital‐based Radio Frequency Quadrupole (RFQ) accelerator BNCT system with fully independent intellectual property rights, is approaching completion, evaluating safety, tolerability, and preliminary efficacy in patients with recurrent or advanced solid tumors.^20^, ^21^ Concurrently, substantial progress has been made in the development of hospital‐based neutron sources, including RFQ linear accelerators and cyclotrons, which are actively pursued across multiple institutions. These technological and clinical advances reflect the rapid maturation of BNCT in China and provide a foundation for broader clinical application and systematic evaluation. Following regulatory approval of its self‐developed BNCT system (NeuPEX) and the boron‐containing therapeutic agent borofalan (^10^B‐BPA), Zhongpeng Medical initiated a registered phase I/II clinical trial at Xiamen Hong'ai Hospital in April 2024. The phase Ia dose‐escalation cohort has been completed, demonstrating favorable safety and tolerability profiles along with preliminary signals of antitumor activity in enrolled patients. The subsequent phase Ib dose‐expansion cohort was initiated in January 2025, with the first three patients having completed treatment. In parallel, the company's ^1^ ^8^F‐labeled BPA PET tracer (NBB‐002), the first BNCT companion diagnostic agent approved for clinical investigation, received Investigational New Drug approval from the Center for Drug Evaluation of the National Medical Products Administration of China. By exploiting the overexpression of L‐type amino acid transporter 1 (LAT1) in tumor cells, this imaging agent allows precise tumor delineation and quantitative characterization of boron biodistribution, thereby facilitating patient stratification, refinement of individualized dose planning, and objective post‐treatment assessment, which are key determinants of enhancing the therapeutic efficacy and clinical outcomes of BNCT.

Despite these advances, BNCT in mainland China faces distinct challenges compared with other radiation therapy modalities. These challenges mainly stem from the unavailability of clinical trial data and need for coordinated technical support across diverse specialized fields, such as physics, medicine, and engineering. To fully establish BNCT as a mainstream treatment option, more comprehensive clinical data are essential to verify its efficacy and safety, and a multidisciplinary approach is necessary.^22^ A particularly significant technical challenge lies in the accurate and real‐time measurement of boron concentration during treatment. This challenge has spurred the development and application of various analytical methods and imaging technologies, including Inductively Coupled Plasma Mass Spectrometry (ICP‐MS), Inductively Coupled Plasma Atomic Emission Spectroscopy (ICP‐AES), PET, and Single‐Photon Emission Computed Tomography (SPECT).^23^ Moving forward, the establishment of standardized technical protocols for neutron equipment, boron agents, and boron monitoring will be crucial for promoting widespread BNCT adoption and addressing these ongoing challenges.

### 1.1 Project background and significance

A 2021 study published in *CA: A Cancer Journal for Clinicians* by Sung et al.^24^ analyzed GLOBOCAN 2020 cancer incidence and mortality estimates from the International Agency for Research on Cancer. Their findings projected that the number of cancer cases would reach 28.4 million globally by 2040, representing a 47% increase from 2020. This burden is expected to disproportionately affect developing nations, with projected increases of 64–95% relative to 32–56% in developed countries. As a developing nation with a substantial aging population, China faces particularly significant challenges in cancer prevention and control. BNCT has emerged as a promising therapeutic modality that offers precise targeting of tumor cells while minimizing damage to healthy tissues. This approach has demonstrated efficacy in treating challenging malignancies, including invasive, recurrent, and metastatic tumors. Clinical studies have shown positive outcomes in various cancer types, including gliomas,^8^, ^12^, ^25^, ^26^ melanomas,^10^, ^20^, ^27^, ^28^ recurrent head and neck tumors,^16^, ^29^, ^30^, ^31^, ^32^, ^33^, ^34^ and cases of lung and liver cancer.^35^, ^36^ The treatment mechanism involves directing a neutron beam—which is harmless to human tissue—at the patient, triggering a nuclear reaction with boron‐containing compounds that have been selectively accumulated in cancer cells. This reaction selectively destroys cancer cells while sparing surrounding healthy tissue, making BNCT a revolutionary approach in precision cancer therapy.

A significant milestone in the development of BNCT technology in China was achieved in October 2006, when a research team from the Institute of High Energy Physics at the Chinese Academy of Sciences, supported by the National Basic Research Program (973 Program), constructed the first high‐current RFQ accelerator in China, achieving a proton energy of 3.5 MeV. Building on this technical foundation, the team initiated a comprehensive BNCT development program in January 2012, encompassing treatment system design and key technology research and development. A breakthrough came in August 2020 with the successful development of China's first domestically produced BNCT experimental device with independent intellectual property rights. The team subsequently conducted in vitro and in vivo evaluations to validate its therapeutic efficacy and began developing a BNCT device for clinical application. This project represents an industrialization effort that utilizes technology related to the China Spallation Neutron Source. The successful realization of this prototype has established a crucial technical foundation for the domestic production and commercialization of medical BNCT systems in China, marking an important advancement in China's cancer treatment capabilities.

As a binary targeted radiotherapeutic modality, BNCT is fundamentally distinct from other emerging precision radiotherapy approaches, such as proton therapy and FLASH radiotherapy, in its mechanism of action, targeting paradigm, and scope of clinical application. These distinctions define a unique therapeutic niche for BNCT within contemporary oncology and are particularly relevant when considering treatment options for refractory malignancies resistant to conventional radiotherapy techniques. Proton therapy exploits the Bragg peak to deliver a conformal dose distribution, sparing proximal normal tissues, making it well suited for deep‐seated tumors such as skull base lesions. However, proton therapy lacks cell‐specific targeting, and its therapeutic efficacy can be compromised by intratumoral heterogeneity and hypoxic microenvironments. In addition, the substantial capital investment (often exceeding 100 million US dollars)^37^ and prolonged fractionated schedules limits its accessibility. FLASH radiotherapy delivers radiation at ultra‐high dose rates (typically >40 Gy/s),^38^ reducing normal tissue toxicity via FLASH effect, which is associated with altered reactive oxygen species dynamics in normal cells. While promising for superficial tumors and in palliative settings, its clinical translation remains constrained by challenges in dose homogeneity for large or irregular tumors, limited penetration depth for deep‐seated lesions, and insufficient data on long‐term safety.

In contrast, BNCT offers two distinctive advantages addressing key unmet clinical needs. First, its relies on the combination of selective ^10^B accumulation and subsequent neutron irradiation, thereby providing a dual‐level targeting mechanism that exceeds the purely physical selectivity of proton or FLASH radiotherapy. This is particularly advantageous for infiltrative tumors with poorly defined margins, such as glioblastoma. Second, the high linear energy transfer alpha particles and recoiling lithium nuclei have path lengths limited to a single‐cell diameter, enabling highly localized cytotoxicity at the cellular level. Importantly, this effect is independent of oxygen availability, allowing BNCT to overcome hypoxia‐associated radioresistance.

Despite these strengths, several practical considerations influence the clinical implementation of BNCT. Clinical deployment depends on access to suitable neutron sources, either logistically constrained nuclear reactors or accelerator‐based systems that require specialized technical infrastructure. In addition, achieving therapeutically relevant intratumoral boron concentrations together with favorable tumor‐to‐normal tissue and tumor‐to‐blood ratios remains a central challenge, underscoring the need for continued optimization of boron delivery agents. Furthermore, in contrast to proton therapy, BNCT lacks widely accepted standards for boron concentration monitoring, treatment planning, and outcome assessment, which complicates interinstitutional comparison and broader clinical adoption. Consequently, BNCT should be viewed not as a replacement for existing precision radiotherapy techniques but as a complementary modality suited to specific high‐priority clinical scenarios, including recurrent gliomas, hypoxic solid tumors, and infiltrative head and neck cancers. Ongoing advances in accelerator‐based neutron sources and next‐generation boron carriers with improved targeting efficiency and bioavailability, are expected to facilitate its integration into multimodal cancer treatment strategies, particularly in contexts where proton and FLASH radiotherapy offer limited benefit.

### 1.2 Principles of BNCT

#### 1.2.1 Operating principle of BNCT devices

The BNCT neutron source consists of four main components: an Electron Cyclotron Resonance (ECR) microwave proton source, low‐energy transport line, RFQ accelerator, and high‐energy transport line. The ECR microwave proton source generates the requisite proton beam for the accelerator system. This proton beam is extracted at high voltage and passes through the low‐energy transport line, where it is accelerated by the RFQ accelerator. The protons are accelerated to a maximum energy of 2.8 MeV before impacting a lithium target for neutron generation through the following nuclear reaction:


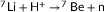




The fast neutrons produced undergo moderation and beam shaping processes before entering the treatment room. Once inside the patient's body, these neutrons interact with previously administered ^10^B atoms accumulated in the tumor. This reaction triggers a nuclear reaction that produces alpha particles and lithium particles, which are heavy ions that effectively destroy cancer cells.

#### 1.2.2 Principle of Neutron therapy

BNCT employs a selective boron delivery agent (e.g., BPA) to achieve effective ^10^B accumulation in tumor cells. ^10^B is selected for its high tumor affinity, superior thermal neutron capture cross‐section compared to biological elements (C, H, O, N), stability, non‐toxicity, and natural isotopic abundance. Upon thermal neutron irradiation of the tumor region, ^10^B within the tumor cells captures neutrons, entering an excited state that decays to produce alpha particles (^4^He) and recoiling lithium nuclei (^7^Li) with high linear energy transfer, destroying tumor cells through the following nuclear reaction:


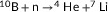




The alpha particles and recoiling ^7^Li nuclei have ranges of approximately 9 µm and 4 µm, respectively, comparable to typical tumor cell dimensions (about 10 µm). This spatial confinement ensures localized cytotoxicity within tumor cells and their immediate vicinity, maximizing therapeutic efficacy while sparing surrounding healthy tissue.

The evolution of boron delivery agents spans three generations. In the 1950s and 1960s, the initial clinical trials of BNCT employed simple boron compounds including boric acid (H_3_BO_3_), sodium tetraborate, and its derivatives. These compounds were selected for their low toxicity, abundant supply, and favorable pharmacological properties. However, treatment outcomes were suboptimal due to a lack of tumor selectivity and limited penetration capability of thermal neutrons.^39^, ^40^, ^41^ Subsequent research by Hatanaka and others rekindled interest in BNCT, leading to the development of second‐generation boron compounds,^42^ notably BPA and BSH. These compounds, however, faced limitations: BPA suffers from low boron content and poor water solubility; while BSH lacks tumor‐targeting specificity and carriers a negative charge hindering cell membrane penetration. To address these limitations, third‐generation BNCT drugs were developed, incorporating various boron‐containing compounds and carrier systems, such as boron‐modified peptides, amino acids, carbohydrate derivatives, porphyrins, nucleosides, and liposomes.^1^

The success of BNCT critically depends on achieving sufficient accumulation of ^10^B in the tumor while delivering adequate thermal neutron doses to induce cytotoxic effects on the tumor cells. Consequently, boron‐containing compounds must therefore meet several essential requirements:^43^ minimal biological toxicity and good water solubility; high tumor uptake, achieving ^10^B concentrations ranging from 20 to 50 µg/g; prolonged retention in cancer cells; rapid clearance from blood and healthy tissues, yielding high tumor‐to‐normal tissue (T/N > 5) and tumor‐to‐blood (T/B > 3.5) ratios.

To enhance the efficacy of BNCT, continuous development of more effective boron delivery agents with improved tumor selectivity and increased ^10^B content remains crucial. Recent reviews on BNCT have provided valuable insights into specific subfields: for example, Dymova et al.^44^ focused on the clinical translation of BNCT for recurrent head and neck cancers, with an emphasis on treatment outcomes and neutron source optimization. Other contemporary reviews have either concentrated on single‐class boron carriers (e.g., only nanoparticles or small‐molecule agents) or limited their scope to specific disease types (e.g., solely brain tumors or melanoma). In contrast, this review distinguishes itself through three core characteristics: first, it adopts an integrated “mechanism–carrier–clinic–technology” framework, systematically linking the structural design of boron‐10 agents to their in vivo pharmacokinetics, therapeutic mechanisms, and clinical application potential; second, it covers the full spectrum of third‐generation boron carriers (from amino acids, peptides, and carbohydrates to multifunctional nanocomposites, Metal‐Organic Frameworks (MOFs), and radiolabeled probes) with critical comparative analysis of their advantages and limitations; third, it incorporates the latest progress in accelerator‐based neutron sources (AB‐BNCT) and real‐time boron monitoring technologies (e.g., ^45^, ^46^, ^47^ BBPA‐PET), establishing a bridge between basic research and clinical translation. By addressing the gaps of previous reviews, such as insufficient integration of carrier design and clinical needs, or lack of critical evaluation of translational challenges, this work aims to provide a comprehensive and forward‐looking overview for researchers in both academia and industry.

## 2 THIRD‐GENERATION BORON DRUGS FOR BNCT

### 2.1 BPA and BSH derivatives

BPA was first synthesized by Snyder and colleagues in 1958.^48^ The capacity of BPA to participate in melanin synthesis, coupled with its preferential uptake by melanoma cells, led to its early application in BNCT for treating cutaneous melanomas.^49^ Yoshino et al. later developed BPA‐F by complexing BPA with fructose, which significantly improved its water solubility at physiological pH, thereby enhancing ^10^B delivery to tumor cells.^50^ The potential applications of BPA expanded considerably when Coderre et al. demonstrated its ability to deliver therapeutic concentrations of ^10^B to various cancer types, including 9L rat glioma.^51^ This breakthrough catalyzed the initiation of high‐grade clinical trials investigating BNCT with BPA‐F as the boron carrier for glioma treatment.^52^, ^53^, ^54^ Subsequently, the therapeutic application of BPA‐F extended to extracranial malignancies, particularly head and neck cancers.^1^ In 2025, Dai et al. further advanced the modification strategy of BPA by developing multifunctional vesicles based on a lipoic acid–boronophenylalanine (LA–BPA) derivative.^55^ These vesicles exhibit reversible self‐assembly under specific pH conditions and target sialic acid on tumor cell surfaces via phenylboronic acid moieties, thereby enhancing tumor‐specific delivery. This innovative design provides a novel approach to overcoming the limitations of BPA and promotes the development of precision and high‐efficiency nanomedicine in cancer therapy.

The development of BSH is closely linked to advances in carbon–borane chemistry. Carborane structures, which typically contain more than 10 boron atoms, enable an effective increase in tumor boron concentration when used as carriers. In 1967, Soloway et al. synthesized Na_2_[^10^B_122_H_12_SH] (BSH), a sulfhydryl derivative of [B_2_H_22_]^2^^−^ as a sodium salt, and demonstrated its specificity for brain tumors in vivo.^56^ Given its high boron content, BSH has attracted significant attention from BNCT clinical investigators, leading to numerous BSH‐BNCT clinical trials for high‐grade gliomas across Japan and other countries.^26^, ^57^

BPA and BSH exhibit distinct mechanistic profiles; BPA primarily targets proliferating tumor cells and achieves higher intracellular boron concentrations, resulting in enhanced tumor cytotoxicity and reduced side effects compared with BSH‐BNCT.^12^, ^58^ The concurrent administration of BPA and BSH promotes more homogeneous intertumoral boron distribution, thereby enhancing BNCT efficacy. Various improvements in the BPA and BSH delivery methods have significantly increased their uptake by brain tumors, thus enhancing BNCT outcomes. These advancements include the use of intravenous mannitol hypertonic solution,^59^, ^60^ focused ultrasound,^61^ to facilitate blood–brain barrier penetration, and direct intertumoral administration via convection‐enhanced delivery.

The advancement of PET technology, particularly ^1^ ^8^F‐labelled ^1^ ^8^F‐BPA, enables visualization of ^10^B distribution in the human body by PET imaging, providing valuable guidance for developing and implementing BNCT treatment regimens.^29^, ^30^, ^62^ After decades of development, only BPA and BSH are currently approved for clinical use, with BPA being the preferred boron carrier for BNCT in patients with high‐grade gliomas and recurrent head and neck tumors. Although these second‐generation boron carriers have limitations, they demonstrated significant therapeutic benefits by extending patient survival, marking a crucial milestone in the evolution of boron delivery agents.

The limitations of BPA, including compromised water solubility, limited boron content, and insufficient tumor retention, have prompted researchers to pursue various structural modifications. Notable advances include the enhancement of water solubility through the formation of borate esters with glucose to facilitate drug delivery.^63^ This advancement rapidly progressed to clinical implementation for treating high‐grade glioma patients, initially in the United States,^64^, ^65^ followed by several clinical trials across Finland,^34^, ^66^ Sweden,^53^, ^67^ and Japan.^12^, ^17^, ^25^, ^68^, ^69^ These extensive clinical investigations demonstrated the superior therapeutic efficacy of BPA compared with BSH.

**FIGURE 1:**
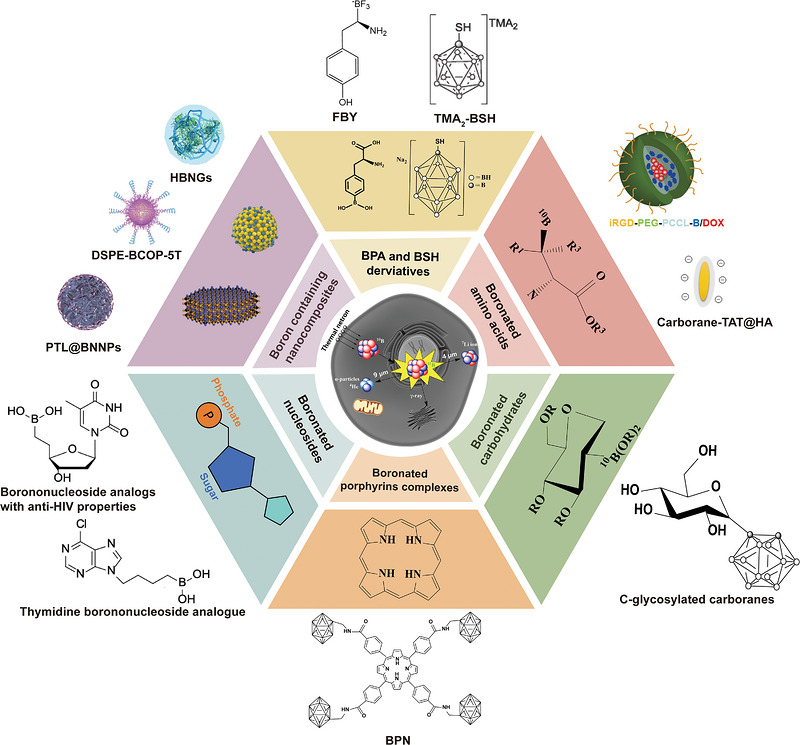
Summary of different types of boron drugs.

**FIGURE 2:**
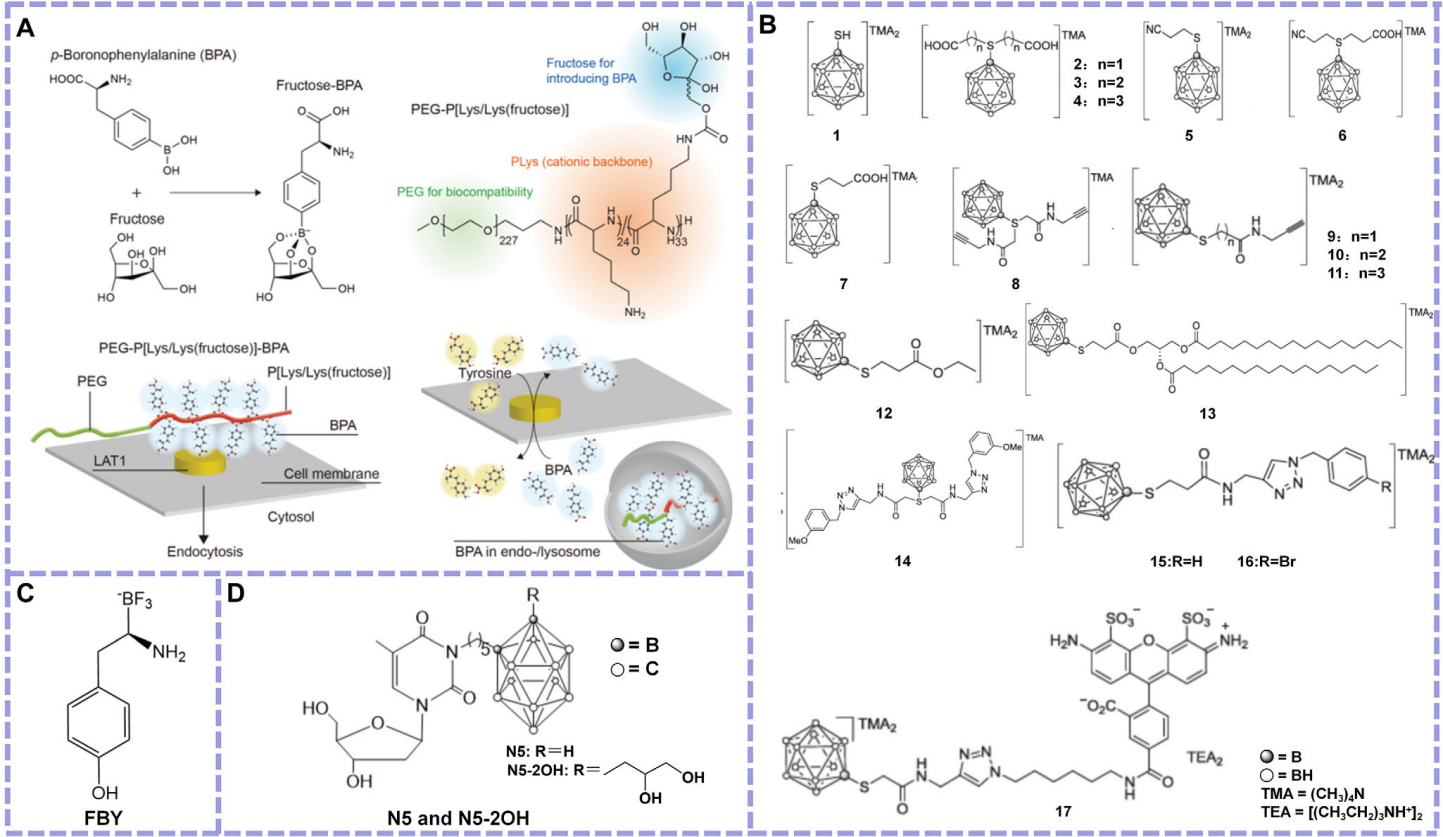
(A) Chemical structures of p‐boronophenylalanine, fructose, fructose‐BPA, and PEG‐P[Lys/Lys(fructose)]; and illustration of the PEG–P[Lys/Lys(fructose)]–BPA complex and its possible interaction with a cell; (B) Chemical structures of mercaptoundecahydrododecaborate (B_12_H_11_SH_2_, BSH) bearing mono‐ and dicarboxyalkyl derivatives; (C) Chemical structure of FBY; (D) Chemical structures of N5 and N5‐2OH.

In 2021, Nomoto et al. reported the development of a complex composed of a fructose‐modified polyethylene glycol‐poly(L‐lysine) block copolymer and p‐boronophenylalanine, designated as PEG‐P[Lys/Lys(fructose)]‐BPA (Table 1, Figures 1 and 2A). This complex enhanced the water solubility of p‐boronophenylalanine through fructose modification, while its polyamine groups facilitated intracellular accumulation and retention within tumor cells. The complex demonstrated significant tumor growth inhibition and exhibited favorable characteristics in mouse models, including efficient renal clearance, specific cellular uptake, and high tumor accumulation.^70^

**TABLE 1:** Comprehensive Comparison of Core Characteristics of Boron Carriers for BNCT.

Boron Carrier Category	Boron Carrier Example	Boron Loading	T/N Ratio	Toxicity/Biocompatibility	Translation Stage	Core Advantages	Main Limitations
First‐generation Small‐molecule Boron Carriers	Boronic acid and its derivatives	∼1 ^10^B / molecule	1.5–2.5	Moderate toxicity, no tumor specificity	Obsolete (only used in early stage)	Simple synthesis, low cost	Lack of selectivity
Second‐generation Small‐molecule Boron Carriers	BPA	∼1 ^10^B / molecule	3–5	Low toxicity, clinically verified safety	Clinical application	Good blood‐brain barrier penetration	Poor water solubility and retention, rapid in vivo clearance
	BSH	12 ^10^B / molecule	1.2–2.0	ow toxicity, no obvious systemic side effects	Clinical application	High boron content	Lack of biological targeting
**Third‐Generation Boron Carriers**							
*BPA/BSH Derivatives*							
BPA Derivatives	BPA‐F	∼1 ^10^B / molecule	‐	Good biocompatibility, no obvious systemic toxicity	Clinical application	Enhances BPA retention	Retention remains poor, rapid in vivo clearance
	(PEG‐P [Lys/Lys (fructose)]‐BPA)	∼1 ^10^B / molecule	5.0–6.0	Good biocompatibility, no obvious systemic toxicity	In vivo research	Reduces BPA efflux; higher binding affinity to BPA than fructose; optimized water solubility; rapid renal clearance	Dependent on LAT1 expression in tumor cells
	LA‐BPA	1.6% ^10^B element	6.7–7.7	Good biocompatibility, no systemic toxicity; mild inflammation only in kidneys/spleen	In vivo research	Strong tumor selectivity; glutathione‐responsive drug release, capable of co‐loading chemotherapeutics; synergistic effect of BNCT and chemotherapy	^10^B content lower than pure BPA; poor storage stability
BSH Derivatives	BSH‐triazole conjugate	12 ^10^B /molecule	‐	Low toxicity, HeLa cell IC_5_₀=27 µM (significantly lower than parent BSH)	In vitro research	Efficient synthesis via click chemistry, flexible structural modification; high intracellular boron accumulation; applicable for fluorescence imaging	Lack of in vivo tumor model validation; targeting dependent on structural modification
	BSH‐lipid conjugate	12 ^10^B /molecule	‐	Good biocompatibility	In vitro research	Self‐assembles into liposomes, suitable for BNCT delivery; high synthesis yield	No in vivo anti‐tumor experiments conducted; lack of toxicity and boron distribution data
*Derivatives of Boron‐Containing Amino Acids*							
Boron‐Containing Amino Acids	ACBC	‐	‐	Good biocompatibility, no obvious adverse reactions	In vitro research	High contrast between tumor and normal brain tissue; distinguishable between recurrent tumors and radiation necrosis; non‐metabolizable, simple modeling	No boron modification achieved; no BNCT‐related in vitro/in vivo experiments; no clear boron loading and tumor accumulation data
	18F‐FBY	∼1 ^10^B /molecule	24.56±6.32	Excellent biocompatibility, no obvious systemic toxicity or adverse reactions	Phase I Clinical Trial	Extremely high metabolic stability, strong tumor specificity; real‐time quantitative boron concentration via ^1^ ^8^F‐PET; distinguishable between high‐ and low‐grade gliomas in Phase I clinical trial	Radiative operation required for synthesis process
Boron‐Containing Amino Acid Lipids	3‐Carboranylthymidine analogs (N5, N5‐2OH)	10 ^10^B /molecule	‐	Good biocompatibility, no obvious systemic toxicity; hydrolytic products are parent drug + natural amino acids, no toxic residues	In vivo research	Significantly improved water solubility, no organic solvent required for solubilization; controllable hydrolysis rate of 5'‐modified prodrugs; suitable for brain tumor delivery	Excessively high stability of 3'‐modified prodrugs, slow release of parent drug; complex hydrolysis steps of disubstituted prodrugs (3',5'), reduced yield of parent drug
Boron‐Containing Peptides	NPY ‐ carborane conjugate	80 ^10^B / peptide molecule	‐	Good biocompatibility, no inherent cytotoxicity	In vitro research	High boron loading; high receptor selectivity	Prone to aggregation in aqueous solution, affecting bioavailability; hydrophobicity not fully balanced
	A6K/BSH peptide nanotubes	12 ^10^B/BSH molecule	Tumor/blood (T/B) = 3.58	Excellent biocompatibility, no cytotoxicity; no obvious organ damage after systemic administration	In vivo research	Simple preparation; enhances intracellular uptake of BSH; strong tumor‐specific accumulation (T/N ratio up to 40); significantly inhibits glioma cell proliferation after neutron irradiation	Lack of active targeting ligands; complex stability dependent on mixing ratio; irregular aggregates formed at improper ratios; no long‐term toxicity and human clinical trials
	Carborane‐TAT@HA nanomicelles	10 ^10^B / o‐carborane molecule	Tumor/blood (T/B) = 3.58	Good biocompatibility	In vivo research	Dual targeting, strong tumor selectivity; tumor microenvironment‐responsive; high stability; significantly reduces non‐specific uptake of TAT, low boron accumulation in normal tissues	Relatively complex synthesis steps; no neutron irradiation anti‐tumor experiments conducted; only validated for breast cancer, unknown applicability for brain tumors
	iRGD‐PEG‐PCCL‐B/DOX nanoparticles	3.81 wt% ^10^B	Tumor/blood = 14.11; Tumor/muscle = 19.49	Excellent biocompatibility; no cytotoxicity of blank carrier	In vivo research	Synergistic therapy of BNCT + chemotherapy; strong tumor penetration; pH‐responsive release of DOX, enhanced efficacy via nuclear localization	Multiple synthesis steps; no in vivo efficacy validation after neutron irradiation
Boron‐Containing Proteins	MID‐BSA	36 ^10^B/BSA molecule	‐	Excellent biocompatibility; no hemolytic toxicity; no damage to blood vessels and components	In vitro research	Flexible preparation; strong tumor selectivity; low organ accumulation, minimal off‐target toxicity; significant tumor inhibition at low doses	Relatively cumbersome synthesis process; dependent on albumin transport; no validation for refractory cancer types
Boronated Carbohydrate Compounds	DGB dendritic glyco‐borane	10 ^10^B/DGBmolecule	‐	Good biocompatibility, no obvious cytotoxicity	In vitro research	Trivalent galactose targets ASGP‐R, multivalent effect enhances affinity; intracellular boron uptake 20 times higher than BSH; improved water solubility via glycosylation	Cumbersome synthesis steps; only validated for hepatoma cells, single applicable cancer type; no in vivo experiments conducted
	MPEG‐b‐P (LA‐co‐D‐Gal‐MPCB)	∼3 wt% ^10^B	Tumor/blood = 25; Tumor/normal liver > 4	Excellent biocompatibility; no systemic toxicity, no obvious damage to liver, kidney and spleen	In vivo research	Galactose targets ASGP‐R, strong hepatoma selectivity; inhibits tumor migration; effectively cleared within 24h	Complex synthesis process; poor applicability for non‐hepatoma; no human clinical trials conducted; not suitable for deep hepatoma
Boronated Nucleotides	Boronated nucleoside analogs (antiviral activity)	4∼10 wt% ^10^B	‐	Strong in vitro cytotoxicity	In vitro research	Dual potential of anti‐HIV‐1 activity and BNCT; easy modification of purine ring for optimized targeting	No in vivo tumor distribution and BNCT anti‐tumor data; higher cytotoxicity than traditional BNCT carriers; no active targeting modification
	Boronated nucleotide analogs (kinase substrate type)	3.5∼4.7 wt% ^10^B	‐	No in vitro cytotoxicity	In vitro research	Flexible saturated linking chain, higher binding affinity to kinases than unsaturated analogs; good biocompatibility; enhanced tumor selectivity via conformational optimization	Low catalytic efficiency; no in vivo boron distribution and efficacy data; no active tumor targeting modification
	Boronated deoxycytidine analogs	3.47% wt% ^10^B	‐	No in vitro cytotoxicity	In vitro research	Phosphorylated by deoxycytidine kinase; induces apoptosis via caspase‐3/7 at high concentrations, no necrotic damage	Low boron content; no in vivo T/N ratio data; no tumor‐killing validation after neutron irradiation
Boronated Porphyrin Derivatives	BTPP	20∼26.5% wt% ^10^B	∼1.4	Moderate toxicity; 10‐19% weight loss in mice after 3‐day infusion; hepatocellular steatosis and thrombocytopenia	In vivo research	Ultra‐high boron loading; porphyrin skeleton mediates tumor accumulation, suitable for PDT+BNCT combination; traceable via fluorescence	Dose limited by hepatotoxicity and thrombocytopenia; high accumulation in normal tissues; poor water solubility
	BOPP	24.5% wt% ^10^B	‐	Toxicity not specified	In vitro research	High boron loading; hydroxyl‐modified side chain enhances flexibility; retains tumor targeting potential, suitable for PDT‐BNCT combination	No in vivo distribution and toxicity evaluation; cumbersome synthesis steps; side chain configuration affects tumor uptake
	VCDP	13.6% wt% ^10^B	‐	Low in vivo toxicity; good water solubility	In vivo research	Natural deuteroporphyrin IX skeleton with strong tumor targeting; balances water solubility and in vivo stability	In vivo T/N ratio and clearance rate not specified; relatively complex synthesis process
	BPN	∼7.6^10^B/molecule	Tumor/muscle = 61.46±20.26; Tumor/blood = 33.85±5.73	Excellent biocompatibility; B16‐F10 cell viability >77.3% (90 µg/ml); no obvious systemic toxicity;	In vivo research	Dual‐modal imaging guidance, real‐time quantitative boron distribution; boron targets cell nucleus, strong tumor‐killing efficiency; tumor boron concentration up to 130 ppm	Relatively complex synthesis process; no human clinical trials conducted
*Nanocomposites*							
Boron‐Containing Nanoparticles	HBNGs	Boron concentration in basic dispersion: 27 ppm	Tumor/blood = 19; Tumor/skin = 3.7	Excellent biocompatibility, no obvious cytotoxicity; no significant weight loss in vivo, no abnormal organ damage	In vivo research	High boron content; high tumor boron accumulation; complete tumor regression after neutron irradiation, no recurrence within three months	Lack of active targeting ligands; no validation for refractory cancer types; complex synthesis process; no human clinical trial data
	o‐Carborane‐loaded PLLGA/PLGA nanoparticles	o‐Carborane content: 4.8–5.6%	Tumor/blood = 5	Excellent biocompatibility, biodegradable; no obvious organ toxicity	In vivo research	High boron content; PLLGA carrier reduces initial burst release of boron, prolongs tumor retention	Lack of active targeting; no in vivo efficacy validation after neutron irradiation; rapid boron release
	PSMA‐targeted PLGA‐b‐PEG nanoparticles	1.86 wt% ^10^B	Tumor/blood = 25; Tumor/muscle < 3	Good biocompatibility, no obvious in vivo toxicity; no organ‐specific accumulation	In vivo research	Theranostic integration; FDA‐approved PLGA‐PEG carrier, high clinical translation potential; prominent targeting selectivity	Extremely rapid boron release, insufficient tumor boron accumulation; poor retention; only validated for prostate cancer一
	Folate‐functionalized boron phosphate nanoparticles	9.8 wt% ^10^B	‐	Good biocompatibility; low cytotoxicity	In vivo research	Improved blood compatibility after functionalization, reduced off‐target toxicity; simple and low‐cost preparation, scalable production	Large particle size may affect tumor penetration; no in vivo quantitative boron accumulation and neutron irradiation efficacy validation
	Folate‐functionalized boron phosphate nanoparticles	Intracellular boron concentration in tumor cells: 246.8 µg/g	Tumor/non‐tumor boron concentration ratio = 15	Good biocompatibility; no obvious damage to blood cells	In vitro research	Tumor boron concentration far exceeds clinical requirements; significantly induces tumor cell apoptosis after neutron irradiation; suitable for NFPAs tumors	Lack of in vivo tumor distribution, T/N ratio and metabolism data; in vivo metabolic clearance mechanism not clarified
	B‐PDA NPs	Peak tumor boron concentration: 29.68±2.23 ppm	Tumor/normal brain = 7.60±1.91; Tumor/blood = 6.83±1.75	Excellent biocompatibility, no obvious organ toxicity	In vivo research	LAT1‐mediated active targeting, blood‐brain barrier penetrable, suitable for glioma treatment; tumor boron accumulation meets clinical requirements, long retention time	Low boron loading, requiring high administration dose; only validated for U87 glioma; particle size affects deep tumor penetration
	PTL@BNNPs	Intracellular boron concentration: 125 ppm	Tumor/blood = 2.71±0.96; Tumor/muscle = 8.32±1.07	Excellent biocompatibility, no obvious dark toxicity; no pathological abnormalities in major organs	In vivo research	Low toxicity; high boron content; PET imaging guidance to optimize irradiation timing; significant tumor inhibition	Additional vitamin C injection required to trigger degradation, increasing treatment steps; dependent on EPR effect, lack of active targeting ligands
	cRGD‐COS‐CB/PTX	6.28 wt% ^10^B	Tumor/blood = 4.38±0.51	Excellent biocompatibility, low toxicity; no obvious organ pathological damage; low hemolysis rate	In vivo research	Dual targeting, strong hepatoma selectivity; synergistic therapy via multiple pathways; boron accumulation far exceeds clinical requirements, long tumor retention time	Potential increased systemic toxicity from combined chemotherapy and BNCT; no quantitative in vivo efficacy validation after neutron irradiation
	T‐Gal‐B‐Cy3@MSN	∼60 wt% ^10^B	Boron delivery efficiency: 40‐50 times that of BSH	Excellent biocompatibility, no cytotoxicity; good water dispersibility	In vitro research	ASGPR receptor‐mediated liver targeting, specific binding to HepG2 cells; dual‐functional with fluorescence imaging; significant tumor killing after neutron irradiation	Only validated for HepG2 hepatoma; lack of in vivo quantitative boron accumulation and T/B ratio data
	B‐MSNs	1.27 wt% ^10^B	‐	Excellent biocompatibility, no obvious in vitro/in vivo toxicity; no organ pathological damage	In vitro research	ACPP peptide activated by tumor microenvironment to enhance penetration; dual‐functional with MRI imaging; kills drug‐resistant cancer stem cells	In vivo tumor boron accumulation and T/B ratio not clarified; no efficacy validation in orthotopic chondrosarcoma model
Gold Nanoparticle‐Based Systems	9SH‐OCB	Cellular boron concentration: 13.5 ppm	‐	Excellent biocompatibility; no obvious cytotoxicity	In vitro study	Thiol‐mediated stable loading of carborane; water solubility can be improved via PEO‐b‐PCL modification	Poor water solubility without modification; difficulty in completely washing boron adsorbed on the cell surface, potentially overestimating accumulation
	9,12SH‐OCB	Cellular boron concentration: 5.75 ppm	‐	Excellent biocompatibility; no obvious cytotoxicity	In vitro study		
	61‐B‐AuNPs	Tumor boron concentration: 217.1±47.1 µg/g	41.05±11.15	Good biocompatibility; stable in serum for 36 h; no obvious organ toxicity	In vitro study	Active targeting via anti‐HER2 antibody (61 IgG); ^1^ ^2^ ^3^I labeling enables SPECT/CT imaging tracking	Increased thyroid uptake after 36 h, possibly due to antibody‐mediated phagocytic degradation; no verification of BNCT efficacy after neutron irradiation
Multifunctional High‐Boron‐Content MOFs Nano‐Cocrystals	MNCs	7.5 wt% ^10^B	Tumor/Normal tissue = 6.20±0.90; Tumor/Blood = 3.80±0.35	Excellent biocompatibility; no hemolytic toxicity; no pathological damage to major organs	In vivo study	Can cross the blood‐brain barrier, suitable for glioma; ^8^ ^9^Zr‐labeled PET + fluorescence dual imaging; stable boron loading without leakage	Only verified in U87 glioma; boron release rate needs further optimization
	BMOFs	Intracellular boron concentration stably maintained at 60 ppm	‐	Good biocompatibility; stable in serum for 36 h; no obvious organ toxicity	In vivo study	Stable boron metabolism, facilitating accurate RBE calculation; RBE reaches 6.78, 4.1‐fold higher than boric acid; compatible with multiple neutron sources	No long‐term in vivo toxicity testing; only verified in glioma, limited cancer type adaptability
Boron‐Containing Carbon Dots	BCD‐Exos	Tumor boron concentration: 107.07±1.58 ppm	Tumor/normal tissue = 5.28±0.29	Excellent biocompatibility, no obvious in vitro/in vivo toxicity; no hemolytic risk; no pathological damage to major organs	In vivo research	Strong targeting; boron carbon dots (BCD) with fluorescence imaging for real‐time tracking; tumor boron accumulation far exceeds clinical threshold	Complex preparation process; limited tracking of deep clinical tumors via fluorescence imaging
Boron‐Containing Liposomes	Carborane phospholipid liposomes	ntracellular boron concentration: 182 µg/10^6^ 4T1 cells	Tumor/muscle = 37±1; Tumor/blood = 4±0.3; Tumor/brain = 62±2	Excellent biocompatibility, no obvious in vitro/in vivo toxicity; no pathological damage to major organs	In vivo research	Co‐loadable with chemotherapeutics/PARP inhibitors for synergistic therapy; ^6^ ^4^Cu‐labeled PET imaging guidance	Complex production process; dependent on EPR effect, active targeting ligands can be optimized
	spd‐closo‐dodecaborate‐encapsulating liposomes	Maximum tumor boron concentration: 242.2 ppm	‐	Excellent biocompatibility, no obvious toxicity; good liposome dispersibility, no aggregation; no pathological damage to major organs	In vivo research	Ultra‐high boron loading; high liposome yield; strong tumor‐killing effect; long boron retention time	Dependent on EPR passive targeting, lack of active targeting ligands
	Trastuzumab‐liposome‐WSA	Intracellular boron concentration: 132 ppm	‐	Good biocompatibility, no obvious cytotoxicity	In vitro research	Long boron retention time; efficient WSA loading via pH gradient, stable boron loading; intracellular distribution suitable for α‐particle killing range	Lack of in vivo tumor distribution and T/N ratio data; no in vivo efficacy validation after neutron irradiation
	EGF‐liposome‐WSA	Intracellular boron concentration: 55 ppm‐	‐	Good biocompatibility, no obvious cytotoxicity; good liposome dispersibility	In vitro research	Significant tumor‐killing effect after neutron irradiation; high boron loading	No ^10^B‐enriched carrier used, limited effective boron concentration; lack of in vivo boron accumulation and long‐term safety data
BN‐R837@PVP		Tumor boron content: 11048 ppm	Tumor/muscle ≈ 200	Excellent biocompatibility, no hemolytic risk; no pathological damage to major organs	In vivo research	High boron content, high delivery efficiency; immunomodulatory synergy with BNCT to inhibit distant metastasis; strong tumor‐killing effect	R837 release mechanism after neutron irradiation needs further optimization
DSPE‐BCOP‐5T		Intracellular boron concentration: 300 ppm	Tumor/muscle = 25.20±3.41; Tumor/blood = 7.46±0.66	Excellent biocompatibility, no obvious cytotoxicity; strong serum stability; no pathological damage to major organs	Preclinical in vivo	Stable COPs carrier structure, no boron leakage; ^6^ ^4^Cu‐labeled PET imaging guidance; T/N ratio enhanced via three‐injection strategy	Complex synthesis process, precise control of polymerization time required
HVJ‐E/p [MPC‐MAAmBO]		9.82×10^6‐7 10^B/particle	‐	Excellent biocompatibility, no cytotoxicity	In vitro research	High membrane fusion capacity of HVJ‐E envelope to promote intracellular delivery; activates anti‐tumor immunity; stable boron loading	Lack of in vivo tumor boron accumulation and T/N ratio data; no BNCT efficacy validation after neutron irradiation; complex large‐scale production
B‐COF@R837		20.55 ^10^Bwt%	Tumor boron concentration: 19.8 ppm	Excellent biocompatibility, no cytotoxicity; no obvious organ damage; strong serum stability	In vivo research	Neutron‐activated framework defects to release R837; synergy of immunity and BNCT; ^8^ ^9^Zr‐labeled PET imaging; inhibits lung metastasis	Complex COF synthesis process, difficult large‐scale production
Boron Cluster Derivatives	Cobalt bis(dicarbollide) derivatives	18 ^10^B/molecule	‐	High biological stability, low toxicity	In vitro research	High boron content; selectivity adjustable via peripheral modification to reduce off‐target effects	No in vivo boron accumulation and BNCT efficacy validation; poor water solubility, requiring excipient assistance
	Gd‐DOTA‐carborane derivative	10 ^10^B/molecule	‐	Good biocompatibility, stable in serum for 24h; no obvious cytotoxicity	In vitro research	Integrated MRI imaging and BNCT boron delivery for real‐time monitoring; simplified synthesis process, high yield	No BNCT efficacy validation after neutron irradiation; only suitable for B16 melanoma, limited cancer type adaptability
	β‐Carboranylethylamine derivatives	10 ^10^B/molecule	‐	Good biocompatibility, stable carborane skeleton, low toxicity	In vitro research	Modular synthesis, adaptable to multiple alkene substrates; convertible to bioactive analogs for "boron delivery + biological targeting" synergy	No cellular/in vivo boron accumulation tests; no clear tumor model adaptability data; no BNCT efficacy validation after neutron irradiation
Boronic Acid Derivatives	PEG‐b‐PDEAS loaded with PBA/PDBA	12 mg ^10^B /kg	‐	Excellent biocompatibility, no cytotoxicity of carrier; strong serum stability; alleviates off‐target hepatotoxicity	In vivo research	pH‐responsive release to avoid drug leakage; multi‐drug compatibility; prolonged blood circulation to enhance tumor accumulation	No BNCT efficacy validation after neutron irradiation; dependent on EPR passive targeting

In BNCT applications, carboranes are commonly employed as boron clusters due to their capacity to form C–C bonds with organic groups and their high boron content, making them potential alternative boron sources. However, these boron clusters often exhibit extremely poor water solubility and severe toxicity.^71^

BSH, compared to carborane‐based boron clusters, offers several advantages including higher boron content, ionic properties, and lower toxicity, making it more suitable for BNCT applications.^72^ The presence of a thiol group in the BSH cluster facilitates the preparation of tumor‐targeting compounds. Consequently, researchers have synthesized various BSH derivatives by conjugating BSH with biomolecules, including porphyrins, nitroimidazoles, sugars, chlorins, and lipids.^73^, ^74^, ^75^, ^76^, ^77^, ^78^, ^79^

Genady et al. successfully functionalized BSH using click chemistry, synthesizing two types of BSH building blocks containing terminally functionalized carboxyl (S‐carboxyalkylthioantimony undecahydro‐closo‐dodecaborate tetramethylammonium salt) or propargyl groups (Figure 2B). These building blocks can be covalently linked to structures used in BNCT treatment.^80^ This methodology enabled high‐yield preparation of diverse bioconjugated‐BSH compounds (Figure 2C) through amidation, esterification, and Cu(I)‐mediated click cycloaddition reactions. The resulting compounds exhibited low cytotoxicity in HeLa cancer cells and, through conjugation with amino or hydroxyl resins, achieved the boron concentrations required for BNCT.

### 2.2 Derivatives of boron‐containing amino acids

Amino acids and short peptide drug carriers have emerged as promising platforms in the development of a new generation of boron agents. Mounting evidence supports novel delivery strategies utilizing amino acids and short peptides containing boronic acid and/or carborane residues. Studies have demonstrated that certain boron‐containing amino acids can achieve remarkable tumor‐to‐normal ratios, reaching as high as 20:1 in murine models.^81^, ^82^ These findings provide compelling support for their potential application in BNCT. Consequently, the synthesis and investigation of novel boron‐containing amino acid compounds represent a direction for critical research to enhance the efficacy of BNCT and broaden its clinical applications.

#### 2.2.1 Boron‐containing amino acids

A research team led by Liu et al at Peking University developed a metabolically stable boron‐derived tyrosine, designated as fluoroboron tyrosine (FBY) (Figure 2D).^83^ Compared with conventional BPA, FBY demonstrates enhanced metabolic stability and tumor specificity, positioning it as a more promising candidate for BNCT applications. The study also established the potential of [^18^F]FBY‐PET imaging for tumor diagnosis and established a correlation between PET images and boron distribution, enabling non‐invasive estimation of boron concentration.

The team subsequently conducted a pioneering in‐human study investigating the safety, biodistribution, and radiation dosimetry of [^18^F]FBY.^84^ The research examined LAT‐1 expression levels in patients with glioma and included six healthy volunteers (three male and three female) who underwent whole‐body PET scans at multiple time points after [^18^F]FBY infusion. Regions of interest in major organs were manually delineated, and time‐activity curves were generated. Dosimetry calculations were performed using OLINDA/EXM software. Additionally, 13 patients with suspected glioma underwent PET/CT scans 30 min post‐injection. Within 7 days of PET/CT imaging, tumors were surgically resected and subjected to LAT‐1 immunohistochemical staining, which was then correlated with [^18^F]FBY‐PET imaging results. Notably, [^18^F]FBY was well tolerated by all healthy volunteers, with no adverse symptoms observed or reported.

Beyond BPA, researchers have investigated various derivatives of boron‐containing natural amino acids, including alanine,^85^, ^86^ phenylalanine,^87^, ^88^, ^89^, ^90^ cysteine,^91^, ^92^, ^93^ and tryptophan.^94^ Compared with natural amino acids, cyclic amino acids exhibit enhanced metabolic stability and prolonged tumor retention time. Additionally, their favorable water solubility contributes to efficient circulation in the bloodstream. In 1988, Aoyagi et al. showed that 1‐aminocyclohexanoic acid could penetrate the blood–brain barrier, highlighting the advantageous lipophilicity of cyclic amino acids.^95^ Subsequently, Hübner et al. and Nichols et al. further confirmed that 1‐aminocyclobutanecarboxylic acid (ACBC) possesses superior tumor specificity relative to BPA, drawing widespread attention to this class of boron carriers.^96^, ^97^

#### 2.2.2 Boron‐containing amino acid lipids

For BNCT targeting brain tumors, two 3‐carboranyl thymidine analogs (3‐CTAs), designated as N5 and N5‐2OH (Figure 2D), were designed. Due to the insufficient water solubility of these parent compounds in preclinical studies, Hasabelnaby et al. developed amino acid ester prodrugs of 3‐CTAs and converted them into salt forms to enhance solubility.^98^ The water solubility of these amino acid ester prodrugs, evaluated in phosphate‐buffered saline, demonstrated a remarkable 48‐ to 6600‐fold increase relative to the parent compounds. Stability assessments of these amino acid ester prodrugs in various media revealed that their hydrolysis rates were primarily dependent on the amino acid type and esterification site.

#### 2.2.3 Boron‐containing peptides

**FIGURE 3:**
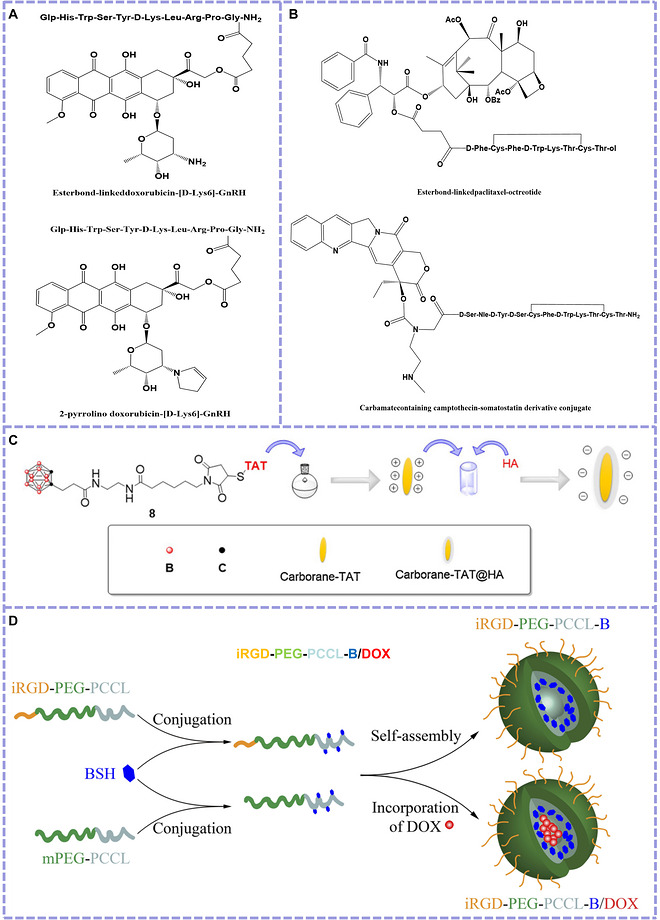
(A) GnRH derivatives: Doxorubicin‐[D‐Lys6]‐GnRH linked by an ester bond; 2‐pyrrolinyl doxorubicin‐[D‐Lys6‐GnRH]; (B) Somatostatin derivatives: Paclitaxel‐octreotide linked by an ester bond; camptothecin‐somatostatin derivative conjugate containing a carbamate group; (C) carborane‐TAT and carborane‐TAT@HA. (inset) Schematic diagram of polymer self‐assembly for targeted delivery of BSH and DOX into cancer cells. Reprinted with permission from Ref.^108^. Copyright 2019, International Journal of Nanomedicine; (D) Schematic illustration of the self‐assembly of the polymer for targeted delivery of BSH and DOX into cancer cells.

Regulatory peptides often have receptors that are overexpressed in tumor cells while showing limited expression in normal tissues. This characteristic makes these peptides and their analogs excellent candidates as drug carriers or targeting moieties, enabling the development of diagnostic and therapeutic agents with enhanced selectivity and reduced off‐target toxicity. In their informative review article “Receptor‐mediated tumor targeting based on peptide hormones,” Mező and Gábor comprehensively evaluated the therapeutic application of gonadotropin‐releasing hormone (GnRH) and somatostatin derivatives (Figure 3A, B) across multiple cancer treatment modalities, including targeted chemotherapy, radiotherapy, photodynamic therapy, BNCT, and cancer diagnostics.^99^ The growing body of preclinical and clinical investigations supporting these approaches presents promising new directions in cancer diagnosis and treatment.

Neuropeptide Y (NPY), a peptide hormone, targets cancer cells through interaction with four human G‐protein‐coupled receptors (hY1R, hY2R, hY4R, and hY5R).^100^, ^101^ Building upon this targeting potential, Worm et al. developed a selective carrier targeting NPY receptors in 2020. Through solid‐phase peptide synthesis, they successfully incorporated a high number of carboranes into NPY receptor‐targeting ligands, thereby enabling targeted delivery to cancer cells.^102^ Their innovative approach yielded multibranched ligands capable of carrying up to 80 boron atoms per molecule while maintaining receptor binding functionality. Importantly, these ligands demonstrated selective cellular uptake in NPY receptor‐expressing cells, establishing the viability of this targeted delivery strategy.

A6K peptide, consisting of six alanine residues and a single lysine residue (AAAAAAK), exhibits lipid‐like self‐assembly properties and forms tubular nanostructures in aqueous solution.^103^, ^104^ The amphiphilic characteristics of this peptide make it particularly effective as a delivery vehicle for negatively charged agents, especially nucleic acid‐based therapeutics.

Harnessing the carrier properties of A6K, Michiue et al. developed a novel boron delivery system through the complexation of A6K with BSH.^105^ The formation of the A6K‐BSH complex is driven by electrostatic interactions between the cationic lysine residue of A6K and the anionic BSH molecule. This elegant approach addresses two critical limitations that have hampered the therapeutic efficacy of BSH in BNCT: poor cellular internalization and non‐specific tissue distribution. The A6K nanotube–mediated delivery system enhances cellular uptake of BSH and improves its tumor‐specific accumulation, thereby potentially increasing the therapeutic index of BNCT. This innovative strategy represents a significant advancement in the development of next‐generation BNCT agents. By harnessing the self‐assembling properties of A6K peptides to overcome the inherent limitations of conventional BSH delivery, this approach opens up new therapeutic possibilities for treating malignant brain tumors and other aggressive cancers amenable to BNCT.

Cell‐penetrating peptides (CPPs), short peptide sequences capable of facilitating intracellular internalization, have emerged as powerful tools for enhancing intracellular drug delivery. These peptides typically function by forming conjugates with therapeutic agents, thereby promoting their translocation across cellular membranes.^106^ Researchers have leveraged this capability to develop an innovative approach for controlling specific localization within cells and optimizing boron delivery for BNCT. Through rational design and synthesis of CPP‐conjugated boron compounds with organelle‐specific targeting capabilities, they achieved both enhanced cellular uptake and precise intracellular localization. This strategic approach induced complex anticancer bioactivities during BNCT, with in vitro neutron irradiation experiments demonstrating significant therapeutic effects, including Adenosine Triphosphate depletion and apoptosis induction.^107^

Quan et al. conjugated the Trans‐Activator of Transcription (TAT) peptide, a well‐characterized CPP, with oxazole to create an amphiphilic boron drug capable of self‐assembling into nanomicelles.^108^ The abundance of surface‐exposed amino groups on the TAT confers positive charges to these nanomicelles, enabling electrostatic interactions with hyaluronic acid (HA) to form core–shell structured nanomicelles (Figure 3C). This core–shell structure not only facilitates targeted delivery in vivo but also incorporates a tumor microenvironment‐responsive feature: controlled shedding of the HA shell in response to tumor‐specific conditions exposes the therapeutic payload to the cellular membrane, thereby enhancing selective uptake by tumor cells.

The RGD (arginine–glycine–aspartic acid) peptide sequence exerts biological functionality through specific interaction with integrins. This interaction initiates a cascade of cellular responses, including proteolytic hydrolysis of proteins, subsequent release of Cysteine–Aspartic acid–Proline–Glycine (CDPG) sequences, and engagement with the NRP‐1 receptor, ultimately facilitating endocytosis and trans‐tissue transport pathways. These mechanisms collectively enhance boron accumulation in tissues.

The iRGD and cRGD peptides, the cyclic RGD variants, have demonstrated significant potential as targeted delivery vehicles for enhancing tumor‐specific drug uptake. In 2019, Qichun Wei et al. at Zhejiang University developed iRGD‐modified polymer nanoparticles for the active targeted delivery of boron and doxorubicin (DOX) in BNCT applications.^109^ Their approach involved synthesizing a ^10^B‐polymer through covalent embedding of ^10^B‐enriched BSH into PEG‐PCCL, followed by surface modification with iRGD peptides and subsequent DOX loading (Figure 3D). This strategic design demonstrated enhanced intracellular delivery of both DOX and boron in A549 cells. in vivo studies revealed favorable pharmacokinetic properties, including extended blood circulation time, enhanced tumor‐specific boron accumulation, and optimal tumor‐to‐normal tissue boron concentration ratios. Building on this success, the research team subsequently synthesized cRGD‐COS‐CB/PTX nanoparticles^110^ and a multifunctional nanoliposome delivery system, DOX‐CB@lipo‐pDNA‐iRGD,^111^ establishing a promising framework for future application of RGD peptides in BNCT.

#### 2.2.4 Boron‐containing proteins

The elevated demand for nucleic acid precursors during malignant cell proliferation presents a unique opportunity for tumor‐specific targeting. This biological characteristic has driven significant research interest in boron‐containing derivatives as potential boron delivery agents. Early investigations focused on the synthesis and evaluation of boron‐containing bases, particularly boron‐modified purines and pyrimidines.^112^, ^113^ However, these compounds exhibited high cytotoxicity and poor hydrolytic stability, which precluded their application in BNCT.^114^, ^115^

Research focus subsequently shifted to boron‐containing nucleosides. Nucleosides can undergo phosphorylation by cellular phosphorylases, generating monophosphates that are retained intracellularly due to their negative charge. The selective expression of cytoplasmic thymidine kinase (TK1), a crucial enzyme in DNA synthesis, in proliferating tumor cells but not in normal tissue, provides a compelling rationale for targeting specificity.^116^, ^117^

Albumin has emerged as another promising platform for boron delivery. Human serum albumin, the predominant monomeric protein in plasma, maintains plasma osmotic pressure and fluid distribution at physiological concentrations of 35–50 g/L.^118^ This 66.5 kDa protein, comprising 585 amino acid residues, exhibits exceptional ligand‐binding versatility, accommodating diverse endogenous and exogenous compounds including fatty acids, bilirubin, uremic toxins, nitric oxide, calcium ions, hormones, and therapeutic agents. The enhanced permeability and retention (EPR) effect, characterized by vascular leakage and compromised lymphatic drainage in malignant tissues, facilitates albumin accumulation in tumors.^119^, ^120^, ^121^ This accumulation is further augmented by increased albumin uptake in rapidly proliferating tumor cells, where it serves as a crucial nutrient source.

Building on this foundation, Kikuchi et al. developed a novel boron carrier: the maleimide‐functionalized closo‐dodecaborate albumin conjugate (MID‐AC).^122^ This compound achieves high boron loading through specific interactions with cysteine and lysine residues under physiological conditions. The synthesis involves conjugating maleimide‐modified closo‐dodecaborate sodium (MID) to the free thiol and lysine residues in albumin via a nucleophilic ring–opening reaction, facilitated by tetrabutylammonium (TBA) azide on the closo‐dodecaborate‐1,4‐dioxane complex. The efficient tumor‐specific accumulation and significant inhibition of colon tumor growth have been experimentally validated, establishing its potential as a promising vehicle for neutron capture therapy.

### 2.3 Boronated carbohydrate compounds

**FIGURE 4:**
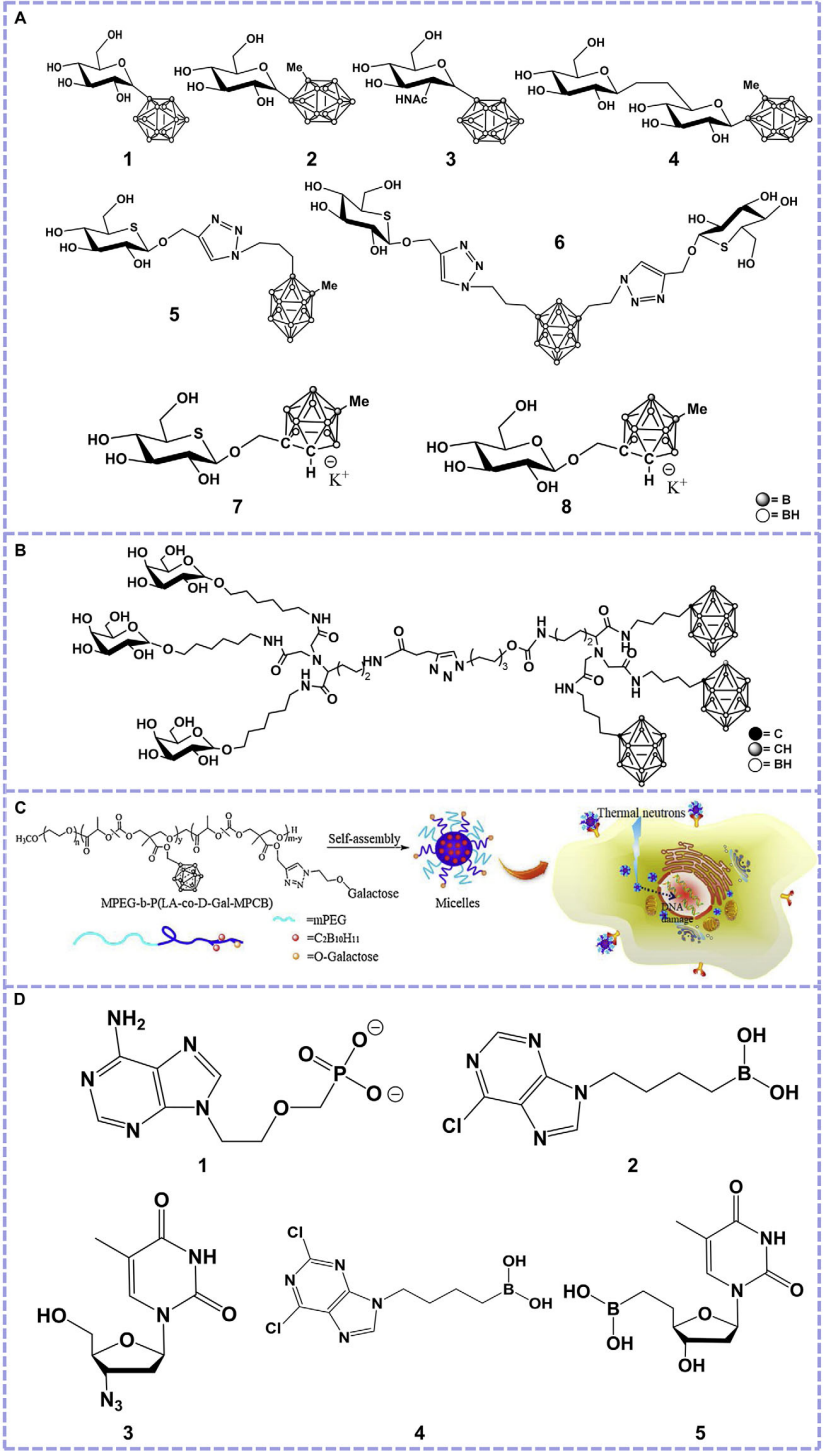
(A) Chemical structures of C‐glycosylated carboranes (1–4) and carboranes containing 5‐thio‐d‐glucopyranose (5–8); (B) Chemical structure of DGB10; (C) Schematic illustration of MPEG‐b‐P(LA‐co‐D‐Gal‐MPCB); (D) Chemical structures of cyclic azidothymidine (AZT) (1), acyclic adefovir (2), two acyclic borono‐nucleoside analogs with anti‐HIV properties (3–4), and thymidine borono‐nucleoside analogue (5).

The field of boron delivery systems experienced a significant advancement in 2003 with the introduction of a novel pre‐spacing strategy that incorporated hydrophilic carbohydrate groups into carborane structures (Figure 4A).^123^ The advantage of this method lies in its versatility, enabling the creation of diverse glycoconjugates with variable spacer types and lengths. The introduction of sufficiently long spacers between the carbohydrate fragments and the bulky carborane cage ensures optimal interaction between the glycoconjugates and endogenous lectins (protein receptors).^124^ This glycoprotein interaction holds particular promise for enhancing the tumor selectivity of BNCT agents. Notably, the recognition of lactose as a ligand for melanoma‐associated lectins has sparked considerable interest in lactose‐based polyhedral boron compounds over the past decade.^125^

In 2012, Lai et al. engineered a multivalent half‐mannosylated borane derivative, named “Dendritic Glyco‐Borane” (DGB) (Figure 4B).^126^ DGB demonstrated superior boron delivery efficacy in HepG2 cells, exhibiting a ten‐fold enhancement in cytotoxicity relative to conventional agents like BSH under neutron irradiation. The optimized molecular architecture enabled specific targeting of liver cancer cells, simultaneously enhancing tumor uptake while minimizing toxicity to normal tissues.

Further advancement came in 2019 when Zhang et al. developed MPEG‐b‐P(LA‐co‐D‐Gal‐MPCB), a PEGylated galactose‐based polymer (Figure 4C).^127^ This polymer self‐assembles into micelles under physiological conditions, demonstrating high selectivity and low cytotoxicity. in vitro studies revealed enhanced uptake in HepG2 cells compared with controls, with BNCT treatment inducing significant cytoskeletal alterations, including reduced cellular migration and DNA double‐strand break–mediated apoptosis. in vivo studies demonstrated a 4.5‐fold increase in tumoral ^10^B concentration relative to BSH, with preferential distribution in hepatic and renal tissues and efficient clearance within 24 h without systemic toxicity. The selective targeting of the asialoglycoprotein receptor (ASGP‐R) by these micelles establishes their potential as effective carriers for hepatocellular carcinoma (HCC)–targeted BNCT.

### 2.4 Boronated nucleotides

Nucleoside drugs have long been effective therapeutics for treating a variety of diseases.^128^, ^129^ For instance, nucleoside analogs such as Zidovudine (AZT) and Adefovir have become established effective medications for treating Human Immunodeficiency Virus (HIV) and Hepatitis B (HBV)^130^ (Figure 4D 1–2), respectively. Synthetic oligonucleotides represent a promising platform for BNCT boron delivery, offering precise interaction with cellular genetic material.^131^, ^132^

In 1996, Chen et al. synthesized acyclic nucleoside boronic acid derivatives^133^ and discovered that two compounds (Figure 4D 3–4) exhibited anti‐HIV activity in vitro, but with high cytotoxicity.

More recently, in 2012, El Amri et al. investigated various borononucleoside analogs as substrates for human nucleoside monophosphate (NMP) kinases.^134^ They found that one of the compounds (Figure 4D 5) acted as a weak substrate for human thymidylate monophosphate kinase, suggesting that such compounds have the potential to participate in nucleotide metabolism.

In this context, Uram and colleagues investigated a boronated 2'‐deoxycytidine derivative, N(4)‐[B‐(4,4,5,5‐tetramethyl‐1,3,2‐dioxaborolan‐2‐yl)]‐2'‐deoxycytidine, demonstrating favorable toxicity profiles in both human U‐118 MG glioma cells and normal human fibroblasts.^135^ The compound exhibited millimolar‐range toxicity in glioma cells, with even lower toxicity in normal fibroblasts, and showed no impact on cellular proliferation at sub‐toxic concentrations. Its demonstrated safety in the *Caenorhabditis elegans* model further supports its potential application in glioblastoma treatment.

### 2.5 Boronated porphyrinl derivatives

Porphyrin compounds have gained significant attention in photodynamic therapy (PDT) due to their selective tumor accumulation, prolonged retention, and unique photochemical properties, establishing themselves as effective clinical anticancer agents.^136^, ^137^, ^138^ The structural versatility of boronated porphyrins, achieved through modulation of alkyl chain length and architecture, further enables enhanced tumoral boron accumulation.

While both BNCT and PDT offer therapeutic benefits, they operate through distinct mechanisms. The neutron beam used in BNCT demonstrates superior tissue penetration compared with the limited depth reached in PDT.^136^ The main advantages of porphyrins—their specific tumor accumulation and capacity to incorporate multiple boron clusters—position boronated porphyrins as ideal candidates for combined BNCT–PDT therapy, potentially enhancing therapeutic efficacy. The clinical success of porphyrin‐based photosensitizers in PDT, coupled with their readiness for boron cluster conjugation, catalyzed the development of porphyrin‐based boron carriers. The early 1990s marked a pivotal moment with the introduction of three key derivatives based on tetraphenyl porphyrin and protoporphyrin: boronated tetraphenylporphyrin (BTPP),^139^ tetrakis‐carborane‐carboxylate esters of 2, 4‐bis‐(α, β‐dihydroxyethyl)‐deuteroporphyrin IX (BOPP),^140^ and 2,4‐divinyl‐nido‐0‐carboranyldeuteroporphyrin IX (VCDP),^141^ establishing the foundation for porphyrin‐based boron carrier development.

The structural diversity achieved through alkyl chain modifications has significantly enhanced tumoral boron accumulation. Notably, the (B_12_H_11_NH_2_)^2−^‐conjugated porphyrin significantly increased ^10^B accumulation levels in HeLa cells, thereby improving the efficacy of PDT.

Additionally, boronated porphyrin derivatives exhibit extensive water solubility, which is a critical requirement for BNCT drugs. The simple yet effective conversion of quaternary ammonium salts into sodium salts has improved the aqueous solubility of these compounds. Recent structural innovations have yielded compounds with superior water solubility and spectral properties, incorporating highly stable, non‐toxic closed boron cluster ions, potentially reducing plasma half‐life and improving therapeutic outcomes.

**FIGURE 5:**
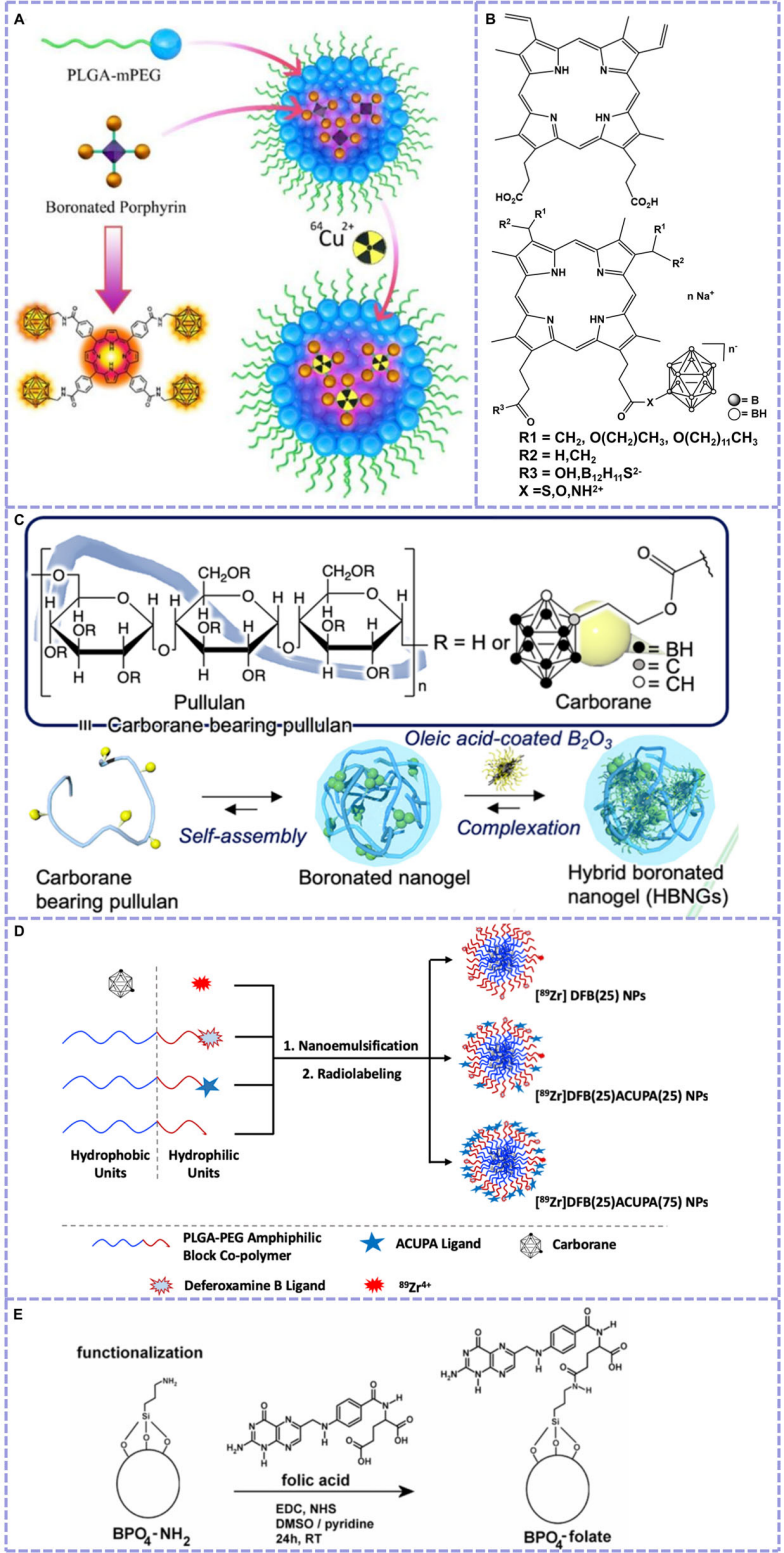
(A) Schematic illustration of imaging‐guided BNCT with BPN; (B) Schematic structures of protoporphyrin IX and sodium salts of boronated protoporphyrin derivatives; (C) Preparation of hybrid boronated nanogel (HBNGs). Reprinted with permission from Ref.^151^. Copyright 2023, Nanomedicine: Nanotechnology, Biology, and Medicine; (D) Representation of PLGA‐PEG Block Copolymer‐Based Amphiphilic Nanoparticles for Targeted Delivery of Boron. Reprinted with permission from Ref.^153^. Copyright 2021, ACS Applied Materials & Interfaces; (E) Schematic representation of covalent labeling of BPO_4_ NPs with folic acid (functionalization).

Liu et al. introduced a novel boron‐doped porphyrin nanocomposite (BPN, Figure 5A), encapsulated within biocompatible polylactic acid‐polyethylene glycol (PLGA‐mPEG) micelles.^142^ This system achieved selective accumulation in tumors while demonstrating reduced toxicity compared with previous boronated porphyrin agents. The integration of fluorescence and PET imaging capabilities facilitated tumor localization and boron concentration determination, enabling optimized treatment planning. Pharmacokinetics studies using ^64^Cu PET imaging led to the development of an optimized multiple small‐dose injection regimen, resulting in enhanced therapeutic outcomes. Post‐neutron irradiation, BPN demonstrated remarkable tumor suppression, with near‐complete tumor regression.

In addition, El‐Zaria's research introduced a series of novel boron‐doped porphyrin derivatives, based on the protoporphyrin IX structure (Figure 5B), incorporating polyhedral borane anions.^74^ The systematic modification of alkyl chain length through ether bond connections to the vinyl group represents a rational approach to developing more efficient boronated porphyrins.

### 2.6 Boron nanocomposites

The emergence of advanced nanotechnology, coupled with increasingly sophisticated drug delivery systems and strategies, has revolutionized modern oncology. Nanomaterial‐based therapeutic platforms have opened new avenues for early detection, precise diagnosis, and personalized cancer treatment.^143^, ^144^, ^145^, ^146^, ^147^ These innovations represent a significant departure from conventional therapeutic approaches and offer renewed promise in oncological interventions.

Nanomedicines possess distinct physicochemical properties that confer several advantages over traditional therapeutic agents, including optimized administration routes, reduced toxicity profiles, and enhanced cost‐effectiveness. Most significantly, these platforms can achieve selective tumor accumulation through both passive and active targeting mechanisms, leveraging the enhanced EPR effect characteristic of solid tumors.^148^, ^149^

Building on these foundational advances in nanomedicine, the development of boron‐enriched nanocomposites with multifunctional properties—such as controlled release, environmental responsiveness, and potent therapeutic efficacy—remains central to advancing BNCT.^150^ Within this context, boron‐containing nanomedicines have emerged as particularly promising boron delivery agents, attracting significant scientific interest. This has led to the synthesis and comprehensive evaluation of multiple classes of boron‐containing nanotherapeutics, establishing their potential as efficient boron carriers for therapeutic applications.^45^, ^46^, ^47^

#### 2.6.1 Boron‐containing nanoparticles

In 2023, Kawasaki et al. designed a novel nanomaterial comprising carbon borane‐modified pullulan nanogels and hydrogenated boron oxide nanoparticles (HBNGs) (Figure 5C).^151^ This platform demonstrated superior boron delivery efficiency compared with the clinical standard boronophenylalanine (L‐BPA)/fructose complex, primarily through enhanced cellular boron accumulation and retention.^152^ The selective accumulation of HBNGs in tumor tissues enabled highly specific boron delivery, meeting clinical demands. in vivo studies demonstrated significant tumor regression following intravenous HBNG administration, with no observable weight loss or tumor recurrence within a three‐month post‐remission period.

Takeuchi et al. developed boron‐loaded nanoparticles synthesized from poly(DL‐lactic‐co‐glycolic acid) (PLGA) and poly(L‐lactic‐co‐glycolic acid) (PLLGA) as BNCT delivery systems in 2017.^153^ The study employed o‐carborane as the boron carrier, with o‐carborane–albumin conjugates serving as controls to develop PLGA and PLLGA nanoparticles with diameters of 100 nm and 150 nm, respectively. The 100‐nm PLLGA nanoparticles achieved notable tumor tissue boron concentrations of 113.9 ± 15.8 µg/g at 8 h post‐administration, maintaining clinically significant concentrations of 20 µg/g without ^10^B enrichment. Nanoparticle formulation with PLGA7510 and PLLGA7510 enhanced tumor boron concentrations by 1.7–3.2‐ and 3.5–4.2‐fold, respectively, relative to o‐carborane–albumin conjugates. Importantly, tumor‐to‐blood boron concentration ratios exceeded 5 at 8–12 h post‐injection, highlighting the suitability of 100 nm PLLGA nanoparticles for BNCT applications.

Subsequently, in 2021, Meher et al. achieved precise targeting of prostate cancer by loading carborane‐containing carriers into poly(lactic‐co‐glycolic acid)‐polyethylene glycol (PLGA‐b‐PEG) nanoparticles functionalized with prostate‐specific membrane antigen (PSMA) ligands (Figure 5D).^154^ This theranostic platform also integrates PET imaging, enabling a companion diagnostic.

In 2014, Achilli et al. conducted a comprehensive biocompatibility assessment of BPO_4_ nanoparticles using mature blood cells (erythrocytes, neutrophils, and platelets) and hematopoietic progenitor cell models.^155^ Their findings demonstrated that BPO_4_ nanoparticles exhibit favorable physicochemical properties and stability in physiological solutions, supporting their potential application in BNCT. The functionalization of BPO_4_ nanoparticles with folic acid (Figure 5E) significantly enhanced their tumor cell uptake, improving cancer cell selectivity—a finding subsequently validated through tumor cell line experiments, establishing a foundation for translational studies and clinical trials.^156^, ^157^, ^158^

Additionally, both unmodified BPO_4_ and BPO_4_‐folate nanoparticles demonstrated minimal adverse effects on blood components. The folate‐functionalized variant showed particular promise in mitigating potential negative impacts on platelet function.^155^

The collective findings establish BPO_4_ nanoparticles as a promising BNCT delivery platform, characterized by excellent biocompatibility profiles, optimal physicochemical properties, selective tumor cell uptake, and minimal hematological complications. These characteristics position BPO_4_ nanoparticles as potential BNCT agents, opening new avenues for therapeutic innovation in cancer treatment.

Non‐functioning pituitary adenomas (NFPAs) present significant therapeutic challenges due to their incomplete surgical resection and high recurrence rates following initial surgery.^159^ Currently, no effective pharmacological interventions exist for the clinical management of NFPAs. While radiotherapy and radiosurgery demonstrate efficacy in preventing tumor regrowth, their application is often limited by severe complications.^160^, ^161^, ^162^

Dai et al. developed a therapeutic approach utilizing folic acid receptor‐mediated carbon nanoparticles containing boron‐10 (^10^B) as delivery vehicles for BNCT. These nanoparticles demonstrated selective uptake by NFPA cells expressing the folic acid receptor, enabling targeted therapeutic effects.^163^ Upon thermal neutron irradiation, treated NFPA cells exhibited significantly decreased viability and increased apoptotic activity, whereas control groups receiving either folic acid receptor–mediated ^10^B‐containing carbon nanoparticles without neutron irradiation or neutron irradiation alone showed no significant alterations in cell viability or apoptotic rates. This study presents a promising therapeutic strategy for aggressive NFPAs resistant to conventional treatments while expanding the potential applications of BNCT in tumor therapy, particularly for benign neoplasms.

**FIGURE 6:**
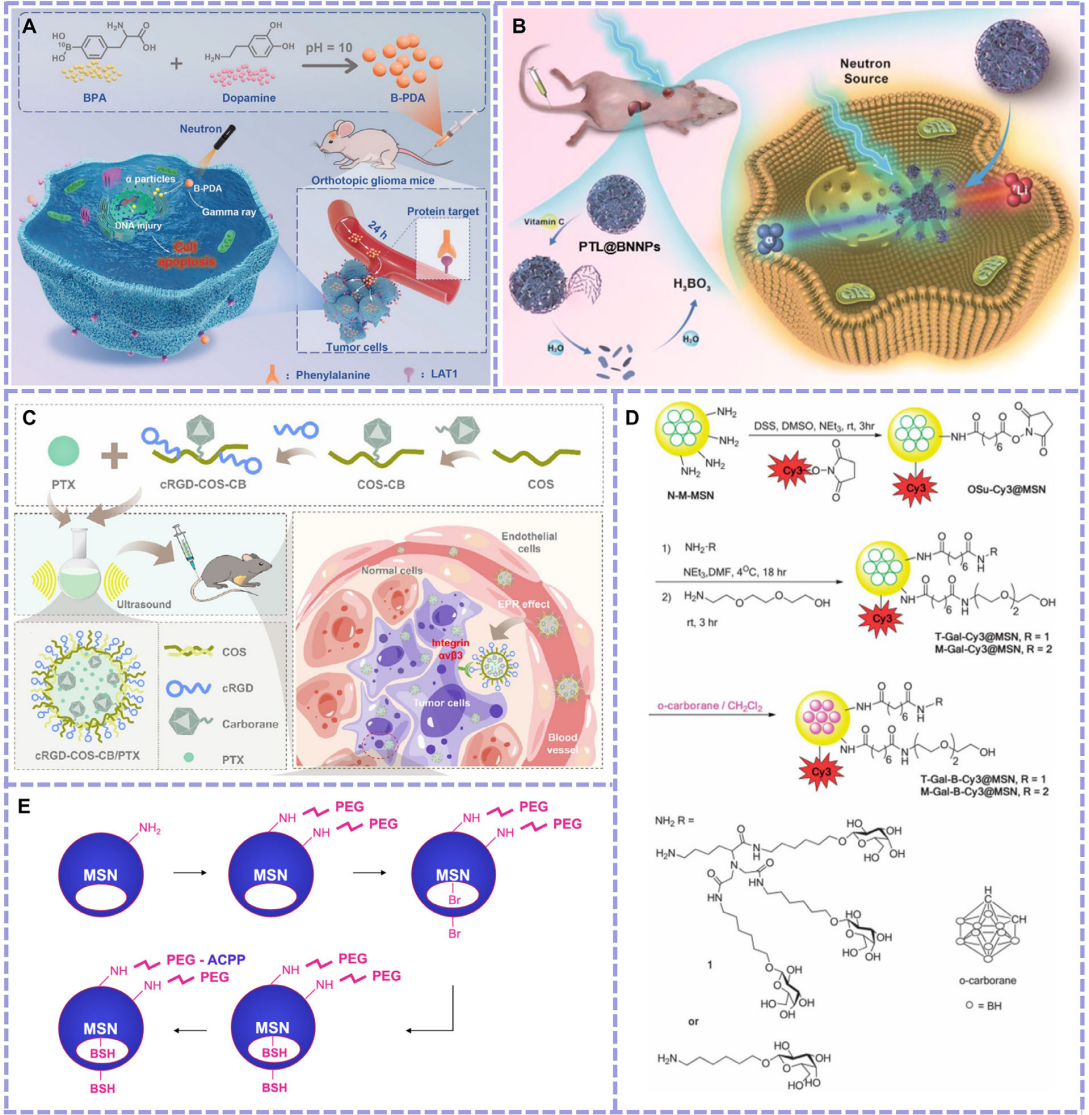
(A) Schematic illustration of the B‐PDA nanoparticle formula and the application of BNCT in a tumor mouse model. Reprinted with permission from Ref.^163^. Copyright 2023, Advanced Functional Materials. (B) Schematic Illustration of PTL@BNNPs Based Boron Neutron Capture Therapy and On‐Demand Degradation. Reprinted with permission from Ref.^168^. Copyright 2019, ACS Nano. (C) Schematic diagram of the structure of nanoparticles (cRGD‐COS‐CB/PTX) and the mechanism of passive and active tumor‐targeting effects of nanoparticles. Reprinted with permission from Ref.^109^. Copyright 2022, Molecular Pharmaceutics. (D) Chemical structure of T‐Gal‐B‐Cy3@MSN. (E) Synthesis of boron‐delivering mesoporous silica nanoparticles.

Qian et al. at Sichuan University developed polydopamine (PDA) nanoparticles encapsulating BPA through nitrogen‐boronate coordination (Figure 6A).^164^ These B‐PDA nanoparticles, when evaluated in an orthotopic xenograft glioma model, showed enhanced tumor‐specific accumulation, prolonged retention within tumor tissues, and superior therapeutic efficacy following neutron irradiation. This groundbreaking research not only presents a novel strategy for BNCT drug design but also validates its exceptional antitumor efficacy in a glioma model.

Boron nitride nanomaterials are structurally similar to carbon nanomaterials, wherein carbon atoms are systematically replaced by alternating boron and nitrogen atoms. These materials primarily include boron nitride nanoparticles (BNNPs) and boron nitride nanotubes (BNNTs). In comparison to their carbon‐based counterparts, boron nitride nanomaterials exhibit superior stability, better biocompatibility, and lower cytotoxicity, making them particularly attractive for biomedical applications, especially drug delivery.^165^, ^166^, ^167^ Their high boron content of up to 50% positions these nanomaterials as promising candidates for boron carrier agents in therapeutic applications.

In a significant advancement, Zhibo Liu et al. developed BNNPs as novel nanocarriers for treating triple‐negative breast cancer. Their approach involved coating BNNPs with phase‐transition lysozyme (PTL), which provided protection against bloodstream hydrolysis while enabling vitamin C–mediated rapid clearance post–neutron capture therapy (Figure 6B).^168^ The coated BNNPs exhibited substantial tumor‐specific boron accumulation while maintaining favorable tumor‐to‐non‐tumor ratios. in vivo studies revealed significant tumor suppression following BNCT treatment, with minimal adverse effects. This strategy successfully leveraged the high boron content of BNNPs while addressing potential toxicity concerns associated with long‐term nanoparticle accumulation through controlled degradability.

Wei Qichun et al. synthesized a novel biocompatible nanoparticle, cRGD‐COS‐CB/PTX (Figure 6C), designed for precise co‐delivery of boron and paclitaxel (PTX) to tumor tissues.^110^ These dual‐functional nanoparticles achieve targeted tumor localization through two complementary mechanisms: passive targeting via the EPR effect and active targeting through surface modification with cRGD peptides. This sophisticated design enables the nanoparticle system to serve as an effective platform for combined radiotherapy and chemotherapy applications.

The discovery of mesoporous silica materials in the 1990s initiated three decades of rapid advancement in the field.^169^ A significant milestone was reached in 2001 with the first report of MCM‐41‐type mesoporous silica nanoparticles (MSNs) as drug delivery systems.^170^ MSNs distinguish themselves among inorganic materials through their unique characteristics: ordered mesoporous structure, tunable pore diameters, extensive surface area, large pore volume, and excellent chemical stability.^169^, ^171^, ^172^, ^173^ Additionally, their surface silanol groups enable functionalization for controlled drug delivery and conjugation with various functional components, including fluorescent imaging molecules and targeting ligands.^174^

In 2013, Lai et al. synthesized T‐Gal‐B‐Cy3@MSN, a multifunctional mesoporous silica nanoparticle (MSN)‐based BNCT agent (Figure 6D).^174^ This agent features a hydrophobic mesoporous architecture capable of substantial o‐carborane encapsulation, while its exterior amine groups facilitate conjugation with trivalent galactose ligands and fluorescent dyes for targeted delivery and imaging. The polarity and hydrophilicity of the galactose ligand enhance aqueous dispersibility while preventing o‐carborane leakage. Moreover, T‐Gal‐B‐Cy3@MSN demonstrated 40–50 times greater boron delivery efficiency relative to BSH, which is the conventional clinical BNCT agent. Cell viability assays demonstrated that T‐Gal‐B‐Cy3@MSN exhibited very low cytotoxicity to HepG2 cells.

By 2020, Vares et al. introduced an innovative boron delivery system utilizing multifunctional fluorescent MSNs (B‐MSNs). These nanoparticles were equipped with activatable cell‐penetrating peptides (ACPP) (Figure 6E) to enhance tumor penetration and carried gadolinium for in vivo magnetic resonance imaging (MRI). ^175^

This innovative boron delivery system represents a significant advancement in BNCT technology, offering enhanced precision through targeted radiation therapy while minimizing collateral damage to healthy tissues. The development presents a promising approach for addressing conventional treatment–resistant cancers and establishes new directions for future oncological therapeutic research.

#### 2.6.2 Gold nanoparticle‐based systems (GNPs)

**FIGURE 7:**
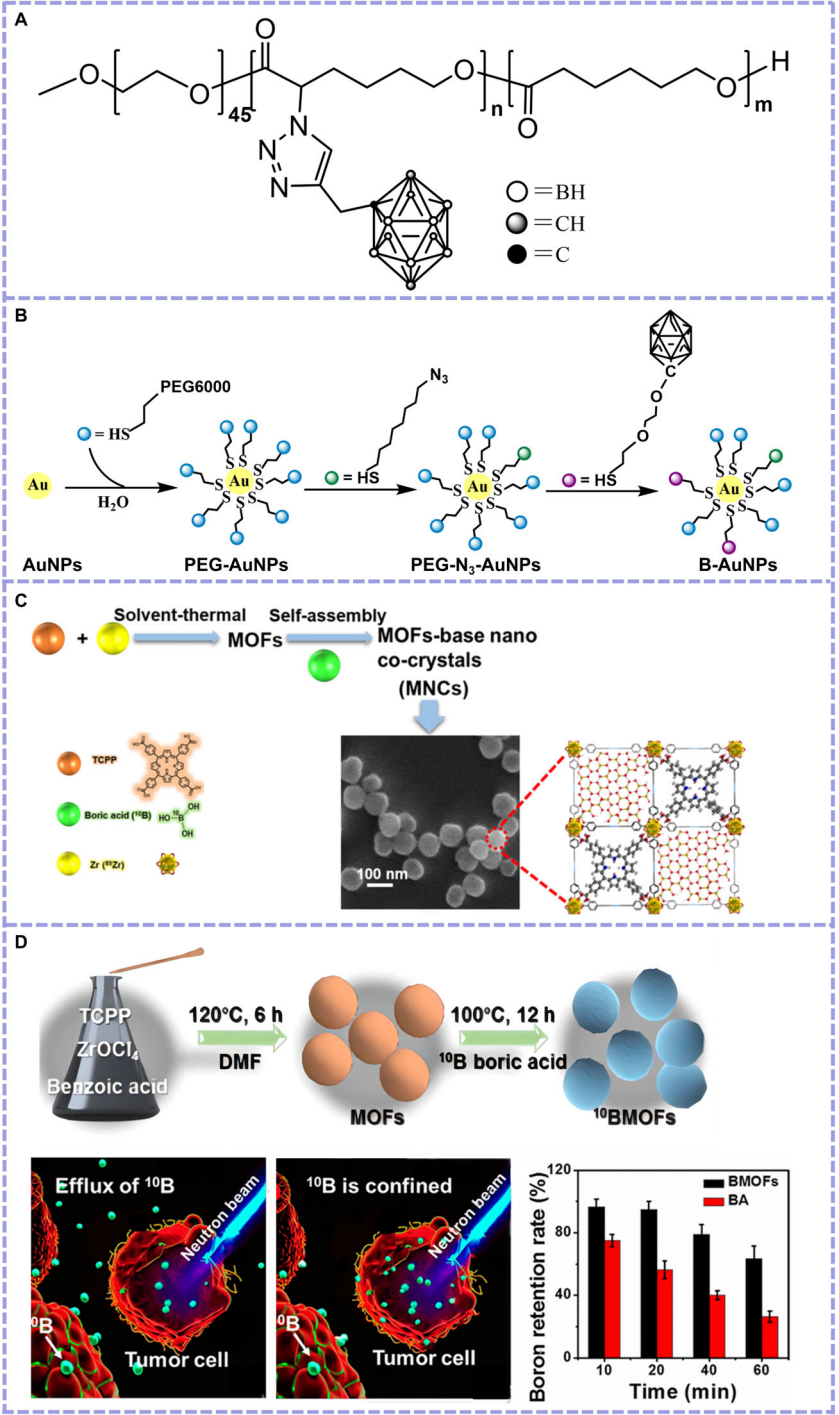
(A) Chemical Structure of PEO‐b‐PCL; (B) Schematic illustration of synthesizing B‐AuNPs. Reprinted with permission from Ref. 176. Copyright 2019, Colloids and Surfaces B: Biointerfaces. (C) MNCs design strategy, function, and brain glioma therapeutic mechanism. Reprinted with permission from Ref. 178. Copyright 2022, Nano Today. (D) Schematic representation of the synthesis and ability to limit intracellular boron efflux of BMOFs. Reprinted with permission from Ref. 179. Copyright 2022, Nano Today.

In 2013, researchers proposed a bottom‐up approach for developing novel boron carriers utilizing framework‐functionalized gold nanoparticles (GNPs).^176^ This innovative design established direct connections between boron atoms from the B_10_C_2_ carborane cage and the gold surface through single or double thiol (SH) bonds. While the resulting GNPs demonstrated stability and low toxicity, their limited aqueous solubility presented challenges for their biological application. This limitation was addressed by coating the carborane‐functionalized GNPs with a specially designed diblock copolymer (PEO‐b‐PCL) (Figure 7A), which simultaneously enhanced hydrophilicity and provided anchoring points to the pre‐functionalized GNPs.

Later, in 2019, Wu et al. developed modified boron–gold nanoparticles with surface‐conjugated boron‐containing compounds (Figure 7B).^177^ The small size of these nanoparticles facilitated tumor penetration through exploitation of relatively large tumor vascular gaps, while their surface modifications enabled targeted drug delivery, enhancing tumor‐specific boron accumulation and subsequent BNCT efficacy. More recently, Pulagam et al. designed multifunctional gold nanorods stabilized with polyethylene glycol (PEG) and functionalized with water‐soluble cobalt bis(dicarbollide) complexes (COSAN).^178^ The in vivo behavior of these nanorods was evaluated through PET imaging using ^64^Cu labeling, demonstrating excellent radiochemical stability and successful tumor accumulation following intravenous administration in a gastrointestinal cancer mouse model.

Multifunctional gold nanorods (AuNRs) labeled with ^64^Cu were successfully synthesized and evaluated as theranostic agents. PET imaging revealed that ^64^Cu‐labeled AuNRs had great radiochemical stability in vivo and demonstrated preferential accumulation in tumor sites following intravenous administration in a murine model of gastrointestinal cancer. The nanorods displayed high biocompatibility and demonstrated dual therapeutic capabilities: localized hyperthermia generation under external stimulation, resulting in cell death within heterogeneous cancer cell spheroids, and effective cancer cell ablation under neutron irradiation. These results suggest that the synthesized nano‐conjugates are promising candidates for combined BNCT and photothermal therapy.

#### 2.6.3 Multifunctional high‐boron‐content MOF nano‐cocrystals

Recent advances in MOFs have demonstrated their exceptional potential as drug delivery systems for BNCT. In 2022, researchers at the Institute of High Energy Physics, Chinese Academy of Sciences, led by Xing et al, developed a nanostructure for precise BNCT treatment of glioblastoma through the synthesis of multifunctional high‐boron‐content MOF nano‐cocrystal structures (Figure 7C).^179^ Their investigation revealed that the zirconium and tetrakis(4‐carboxyphenyl)porphyrin (Zr‐TCPP) MOF exhibited remarkable stability and efficient boric acid loading capability, forming nano‐cocrystal structures (MNCs) characterized by superior biocompatibility, stability, and targeting efficiency. Additionally, the MNCs exhibited dual imaging capabilities through intrinsic fluorescence and zirconium isotope (^89^Zr) positron emission, enabling real‐time in vivo tracking. This development represents a significant advancement in boron carrier technology in glioblastoma treatment, with comprehensive validation of precise in vivo localization and therapeutic efficacy.

Building upon this foundation, in late 2023, the team advanced their research by developing boron‐containing MOF nanoparticles (BMOFs) (Figure 7D) for precise control of intra‐tumoral boron isotope concentrations.^180^ Studies with U87‐MG cells demonstrated that BMOFs maintained stable intracellular boron levels, facilitating accurate determination of the relative biological effectiveness (RBE) of BNCT. Monte Carlo simulations revealed an RBE value of 6.78 for BMOFs, representing a 4.1‐fold increase relative to the conventional small‐molecule boron compounds such as boric acid. The enhanced performance of BMOFs was attributed to their ability to reduce boron clearance through selective accumulation in target cells, resulting in improved therapeutic efficacy.

These findings provide crucial theoretical and experimental foundations for accurately assessing the biological effects of BNCT and establishing a framework for developing personalized BNCT treatment protocols. The demonstrated advantages of BMOFs over traditional small‐molecule boron carriers represent a significant step toward optimizing BNCT outcomes in clinical applications.

#### 2.6.4 Boron‐containing carbon dots

Boron‐containing carbon dots (BCDs) are novel drug delivery systems characterized by their excellent water solubility and favorable optical properties that enable real‐time monitoring of ^10^B distribution in both in vitro and in vivo settings. Xing et al have designed and synthesized a new type of BCDs ^181^ prepared from d‐glucose and p‐hydroxybenzoic acid (BPA) precursors. Chemical characterization confirmed successful boron incorporation into the carbon dot structure. To enhance therapeutic efficacy, the research team encapsulated BCDs within exosomes (Exos), achieving efficient blood–brain barrier penetration and preferential accumulation in glioblastoma tissue with an optimal tumor‐to‐normal ratio. In BNCT, this system successfully facilitated precise intracellular boron localization, ensuring perfect alignment between boron presence and neutron exposure. The remarkable therapeutic efficacy of this approach was demonstrated by the achievement of a 100% survival rate in glioblastoma‐bearing mice, highlighting its significant promise for clinical translation.

#### 2.6.5 Boron‐containing liposomes

Liposomes, predominantly composed of lipid components such as phospholipids, represent a versatile platform for targeted drug delivery in BNCT. These structures offer multiple advantages: the capacity for conjugation with targeting moieties, enhancing tumor specificity beyond the EPR effect, and efficient encapsulation of boron compounds for targeted ^10^B delivery to tumor cells. The targeting capabilities of liposomal carriers eliminate the requirement for tumor‐specific targeting properties in the encapsulated boron compounds themselves.

Liposomal boron encapsulation typically follows two primary strategies: (1) encapsulation of hydrophilic boron compounds within the vesicle; and (2) incorporation of lipophilic boron compounds within the lipid.^182^ The widespread application of liposomes as pharmaceutical carriers stems from their ability to improve drug stability, bioavailability, and targeting efficiency. These advantageous properties have established liposomes as effective delivery vehicles for active boron compounds in BNCT applications.^183^, ^184^, ^185^

One such example is boron‐containing liposomes synthesized with carboranyl phosphatidylcholine, designed for the combination therapy of BNCT and chemotherapy. Both computational simulations and experimental validation demonstrated the exceptional stability of these boron liposomes. Research conducted by Liu et al. utilized ^64^Cu‐labeled boron liposomes for PET imaging, showing high tumor specificity, prolonged retention times, and minimal uptake in normal organs.^186^ Interestingly, they demonstrated that neutron‐irradiated boron liposomes effectively inhibited tumor growth, and the encapsulation of chemotherapeutics, particularly poly (ADP‐ribose) polymerase 1 (PARP1) inhibitors, further improved treatment outcomes. These findings establish boron liposomes as promising multifunctional platforms for chemo‐radiotherapeutic treatment of malignant tumors, offering potential advantages for combined‐modality cancer therapy.

The exceptional utility of liposomes as drug delivery vehicles is particularly evident in their biomedical applications. Their effectiveness is governed by key parameters, including liposome size, surface charge, and drug incorporation methodology, which critically influence carrier–target interactions at cellular and tissue levels. Furthermore, liposomes enhance drug stability in vivo while maintaining excellent biocompatibility.

**FIGURE 8:**
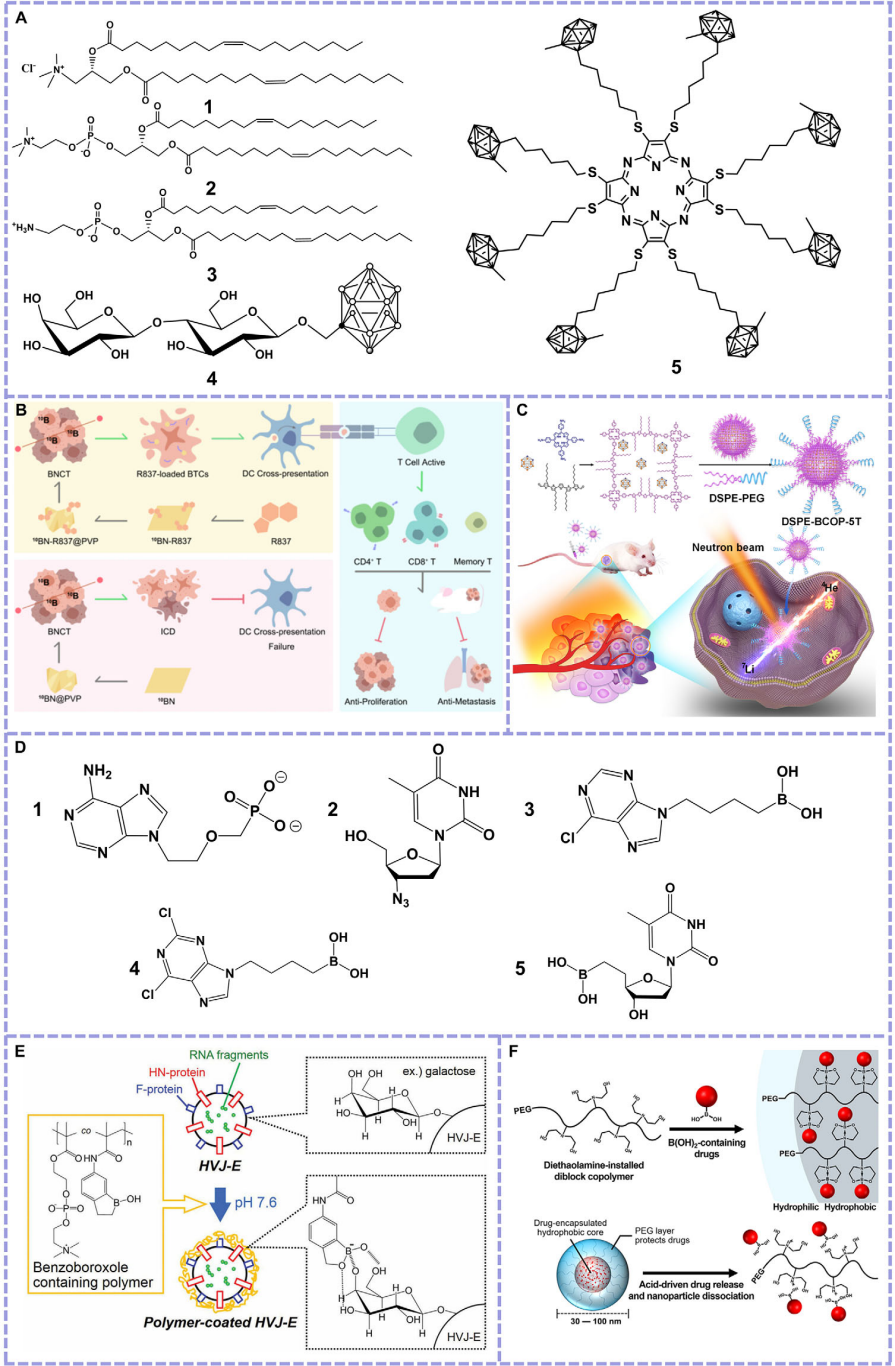
(A) The chemical structures of liposomes (1–3) and novel boron compounds (4–5); (B) ^10^BN‐R837 @PVP constructed by loading R837 on BN nanosheets. Reprinted with permission from Ref. 191. Copyright 2023, Nano Today. (C) Schematic illustration of the fabrication of DSPE‐BCOP‐5T for BNCT. Reprinted with permission from Ref. 192. Copyright 2020, ACS Applied Materials & Interfaces. (D) Schematic illustration of Boron‐Incorporating HVJ‐E. Reprinted with permission from Ref. 197. Copyright 2019, Science and Technology of Advanced Materials. (E) Encapsulation of boronic acid–containing drugs into the nanoparticle and schematic structure of the nanoparticle, and pH‐responsive release of boronic acid‐containing drugs. Reprinted with permission from Ref. 90. Copyright 2021, Acta Biomaterialia.

In the context of BNCT, surface charge modification of liposomes has been recognized as a crucial determinant of therapeutic efficacy. Studies have demonstrated that positively charged liposomes (DOTAP/DOPC/DOPE) (Figure 8A 1–3) exhibited enhanced cellular uptake, attributed to favorable electrostatic interactions with negatively charged cell membrane.^187^ Investigation of these delivery systems using novel boron compounds (LCOB and H_2_PzCOB) in DHD/K12/TRb rat colon cancer and B16‐F10 murine melanoma xenografts (Figure 8A 4–5) revealed remarkable efficacy, with cationic liposomes achieving a 30‐fold increase in intracellular ^10^B concentration compared to conventional BPA. These findings provide valuable insights for developing optimized liposomal boron carriers to achieve higher tumor‐to‐normal tissue boron concentration ratios. Tachikawa et al. developed spermidinium closo‐dodecaborate‐encapsulating liposomes. The innovative use of spermidinium as a counterion for closo‐dodecaborates facilitated both the preparation of high‐boron‐content liposome solutions and efficient tumor‐specific boron delivery.^188^ This novel boron carrier system, offering enhanced boron content compared to traditional compounds, presents potential advantages for improving BNCT outcomes. Their comprehensive evaluation of various closo‐dodecaborates and their cytotoxicity profiles against colon cancer cells has generated valuable data for the continued development of high‐boron‐content liposomal systems.

In 2003, Wei et al. at Zhejiang University developed a new boron‐containing liposome conjugated to Trastuzumab for targeting HER‐2 receptor‐positive tumors.^189^ These Trastuzumab‐conjugated liposomes loaded with water‐soluble boronated anthracene (WSA) demonstrated specific binding, uptake, and internalization in SK‐BR‐3 cells. Building on this success, in 2005, the team developed EGFR‐targeted liposomes incorporating boronated acridine for glioma treatment.^190^ These targeted constructs exhibited exceptional boron delivery efficiency, with subsequent neutron irradiation in BNCT yielding marked cytotoxic effects. Experimental data revealed a ten‐fold enhancement in cytotoxicity compared to neutron irradiation alone, with theoretical calculations suggesting potential cytotoxicity improvements of several orders of magnitude. To further advance glioblastoma treatment strategies, the team developed a multifunctional nanoliposome delivery system. This system utilizes the nuclear targeting properties of DOX to facilitate precise intranuclear boron delivery to the nuclei of tumor cells, enhancing therapeutic efficacy. Its therapeutic efficacy was further augmented through targeted modulation of the CD47‐SIRPα immune checkpoint pathway using CRISPR‐Cas9 technology. in vivo studies demonstrated significant improvements in survival rates, reduction in cancer stem cell populations, and enhanced overall prognosis in murine tumor models. This pioneering research establishes a novel paradigm for combining BNCT with immunotherapy, providing robust theoretical and experimental foundations for advanced glioblastoma treatment strategies.^111^

These investigations collectively demonstrate the exceptional potential of liposomes as carriers for BNCT‐active compounds. Through optimization of surface charge and other physicochemical properties, liposomal delivery systems achieve enhanced drug accumulation in tumor cells. Their superior performance compared with conventional agents, BPA and BSH, establishes liposomes as promising platforms for next‐generation BNCT therapeutics, offering valuable insights for the continued development of more efficacious boron delivery systems and BNCT drug candidates.

#### 2.6.6 2D boron nitride nanosheets

Two‐dimensional boron nitride nanosheets (BNNS) have emerged as promising nanomaterials for biomedical applications, particularly in drug delivery for combined BNCT and chemotherapy.^191^ Their exceptional specific surface area facilitates substantial drug loading capacity, presenting extensive opportunities for therapeutic delivery applications.

Xing Gengmei et al. developed an innovative nanocomposite, BN‐R837@PVP, comprising BNNS functionalized with the immune adjuvant R837 and stabilized with polyvinylpyrrolidone (PVP) (Figure 8B).^192^ This nanodrug was designed to be internalized by tumor cells, delivering R837 directly into the cells. Upon neutron irradiation, it triggers immunogenic cell death, enhancing the efficacy of antitumor immunotherapy while minimizing systemic exposure.

The BN‐R837@PVP nanodrug demonstrated significant advantages by combining efficient tumor targeting and enhanced drug accumulation with stimulation of antitumor immune responses through cross‐presentation. This dual‐action approach effectively inhibits both tumor growth and metastasis. The successful integration of BNCT with immunotherapy in this system represents a promising strategy for clinical translation in cancer treatment.

#### 2.6.7 Conjugated carborane‐polymer carriers (BCOPs)

Liu et al. at Peking University introduced Conjugated Carborane‐Polymer Carriers (BCOPs) as novel carborane carriers (Figure 8C).^193^ These BCOPs were synthesized through a Schiff base condensation reaction, with subsequent alkyl chain engineering and size optimization to enhance their solubility in solvents. The resulting BCOP‐5T was further successfully modified to form stable aqueous nanoparticles suitable for diagnostic applications with PET imaging.

#### 2.6.8 Hemagglutinating virus of japan‐envelope nanomaterials

Hemagglutinating Virus of Japan‐Envelope (HVJ‐E) nanomaterials are envelope nanoparticles derived from hemagglutinating virus of Japan. They possess potent membrane fusion capabilities and are able to integrate genes, proteins, drugs, and other substances for safe tumor cell–targeted delivery. Functionalized HVJ‐E with therapeutic payloads have demonstrated potent capability to stimulate antitumor immune response, broadening their therapeutic potential across multiple oncological applications.^194^, ^195^, ^196^ In BNCT applications, despite the inherent potential of HVJ‐E as a nanoboron carrier, Yoneoka and colleagues advanced its application by developing polymer‐modified HVJ‐E envelopes. The resulting HVJ‐E/Cy5‐p[MPC‐MAAmBO] nanoparticles (Figure 8D) achieved high boron loading capacity while effectively suppressing hemolysis. This biocompatible, low‐toxicity platform represents a significant advancement in BNCT delivery systems.^197^ The integration of HVJ‐E nanoparticles with boron‐containing polymers, achieved through straightforward polymerization and surface modification strategies, has yielded biocompatible polymer‐coated constructs that combine high boron loading capacity with effective hemolysis inhibition. These characteristics, along with their superior fusion capability and tunable surface properties, establish HVJ‐E nanomaterials as promising candidates for next‐generation BNCT delivery platforms.

#### 2.6.9 B‐covalent organic framework (COF) (Boron capsules)

Liu et al. designed a neutron‐activated boron capsule (B‐COF),^198^ utilizing a COF as the delivery platform. This boron capsule combines BNCT with controlled release of immune adjuvants to elicit robust antitumor immune responses. The B‐COF is characterized by its high specific surface area and periodic framework, enabling efficient drug loading and controlled release kinetics while maintaining exceptional biological stability for in vivo applications. The capsule demonstrates efficient cellular internalization through endocytosis, followed by targeted cytoplasmic distribution within tumor cells. Experimental validation, including PET imaging, confirmed its in vivo biodistribution and therapeutic efficacy, particularly its capacity to enhance tumor immunological responses.

### 2.7 Boron cluster derivatives

Boron clusters are polyhedral structures composed of boron atoms, known for their unique chemical stability and diversity. Unlike carbon‐based compounds that typically form chains or rings, boron atoms invariably form clusters due to their lower number of electrons.^199^ These structures, including boranes, carboranes, and metallacarboranes, exhibit remarkable stability and low chemical reactivity, properties that have attracted significant interest in medical applications, particularly in BNCT and radiographic imaging.

In 2012, Kaplánek modified boron cluster compounds by adding positively charged functional groups to their surface, enabling hydrophobic interactions and atypical hydrogen bonding with proteins.^200^ This modification enhanced both stability and biocompatibility within biological systems. These modified boron clusters demonstrated promise in BNCT applications through their ability to engage in molecular recognition with tumor‐associated proteins, thereby improving tumor cell selectivity. Further advancement was achieved by Toppino et al., who developed a bifunctional MRI/BNCT probe based on carboboranes.^201^ This nanoparticle, designed to target tumor cells through low‐density lipoprotein (LDL) binding, achieved significantly enhanced intratumoral boron concentrations, thereby improving BNCT therapeutic efficacy. This modification of boron cluster analogs, especially through the incorporation of charged functional groups or integration with nanomaterials, has demonstrated substantial improvements in both biological stability and specificity, significantly expanding their potential in cancer therapy applications.

Recent years have witnessed significant expansion in boron cluster chemistry research. In 2025, Paulus et al. developed a novel carborane‐based bifunctional reagent that enabled one‐step synthesis of β‐carboranylethylamines through energy transfer (EnT) catalysis.^202^ This innovative approach utilizes photocatalytically generated carboranyl radicals to efficiently construct bioactive β‐carboranylethylamine analogs, offering enhanced possibilities for both BNCT and pharmaceutical development. Furthermore, these carborane derivatives can be subsequently modified to generate azide groups suitable for click chemistry, thereby significantly expanding their applications in bioconjugation and targeted therapy.

These advancements have not only deepened the fundamental understanding of boron cluster chemistry but also established a robust foundation for its medical applications. By integrating Kaplánek's functional group modification strategies with Paulus's efficient synthetic methodology, the therapeutic potential of boron cluster compounds in tumor‐targeted treatment has been further unlocked. The synergistic combination of these approaches demonstrates remarkable promise for developing next‐generation boron delivery systems with improved specificity and efficacy.

### 2.8 Boronic acid derivatives

Boronic acids represent a class of compounds with great potential in biomedical applications. Characterized by a boron atom with an empty p‐orbital, these compounds function as potent electrophilic Lewis acids,^203^, ^204^ readily forming coordinate bonds with nucleophilic agents in biological systems.^205^ Their distinctive chemical properties, including a notable pKa within the weakly acidic to neutral pH range, confer exceptional sensitivity and reversibility to pH changes, rendering them particularly suited for applications such as enzyme inhibition and targeted drug delivery.^206^, ^207^ The nuclear fission properties of boronic acids have positioned them as promising candidates in BNCT, while their versatile chemical characteristics have facilitated widespread applications in drug development, including enzyme inhibitors, cancer‐targeted therapeutics, and diagnostic agents.^208^, ^209^, ^210^, ^211^

To address the clinical limitations of boronic acid derivatives, such as bortezomib, Kim et al. developed a supramolecular polymer nanoparticle delivery system. Leveraging the stable and pH‐sensitive coupling between boronic acids and diethanolamine‐based polymer carriers, this approach enables robust encapsulation and targeted release of boronic acid derivative drugs (Figure 8E). The method has demonstrated efficacy across multiple boronic acid derivatives, including benzo[b]thiophene‐2‐boronic acid, phenylboronic acid, and p‐phenylenediboronic acid, effectively mitigating challenges of low water solubility, rapid excretion, and non‐specific diffusion.^212^

Although many third‐generation boron agents have achieved several‐fold improvements in antitumor efficacy compared with BPA in preclinical models, multifunctional nanocarrier‐based delivery systems, including metal–organic framework nanocrystals, targeted liposomes, and peptide‐functionalized nanoparticles, have emerged as particularly promising owing to their capacity for high boron payloads, compatibility with theranostic integration, and potential to support combination treatment strategies. By comparison, small‐molecule boron compounds are generally limited by low boron content and insufficient active tumor targeting, whereas porphyrin‐based derivatives remain challenged by poor aqueous solubility and concerns regarding systemic toxicity. Despite these encouraging preclinical advances, none of the leading candidates has progressed to late‐stage clinical evaluation. This translational gap is largely attributable to several persistent barriers, including difficulties in achieving reproducible large‐scale nanocarrier manufacturing, the absence of comprehensive long‐term biocompatibility and safety data, the intrinsic complexity of BNCT clinical trial design arising from its binary treatment paradigm, the limited predictive value of existing animal models for the human tumor microenvironment, and the lack of standardized criteria for assessing boron agent performance. Collectively, these factors continue to impede the effective screening, optimization, and clinical translation of next‐generation boron delivery systems.

## 3 RADIOLABELED BORON PROBES

**FIGURE 9:**
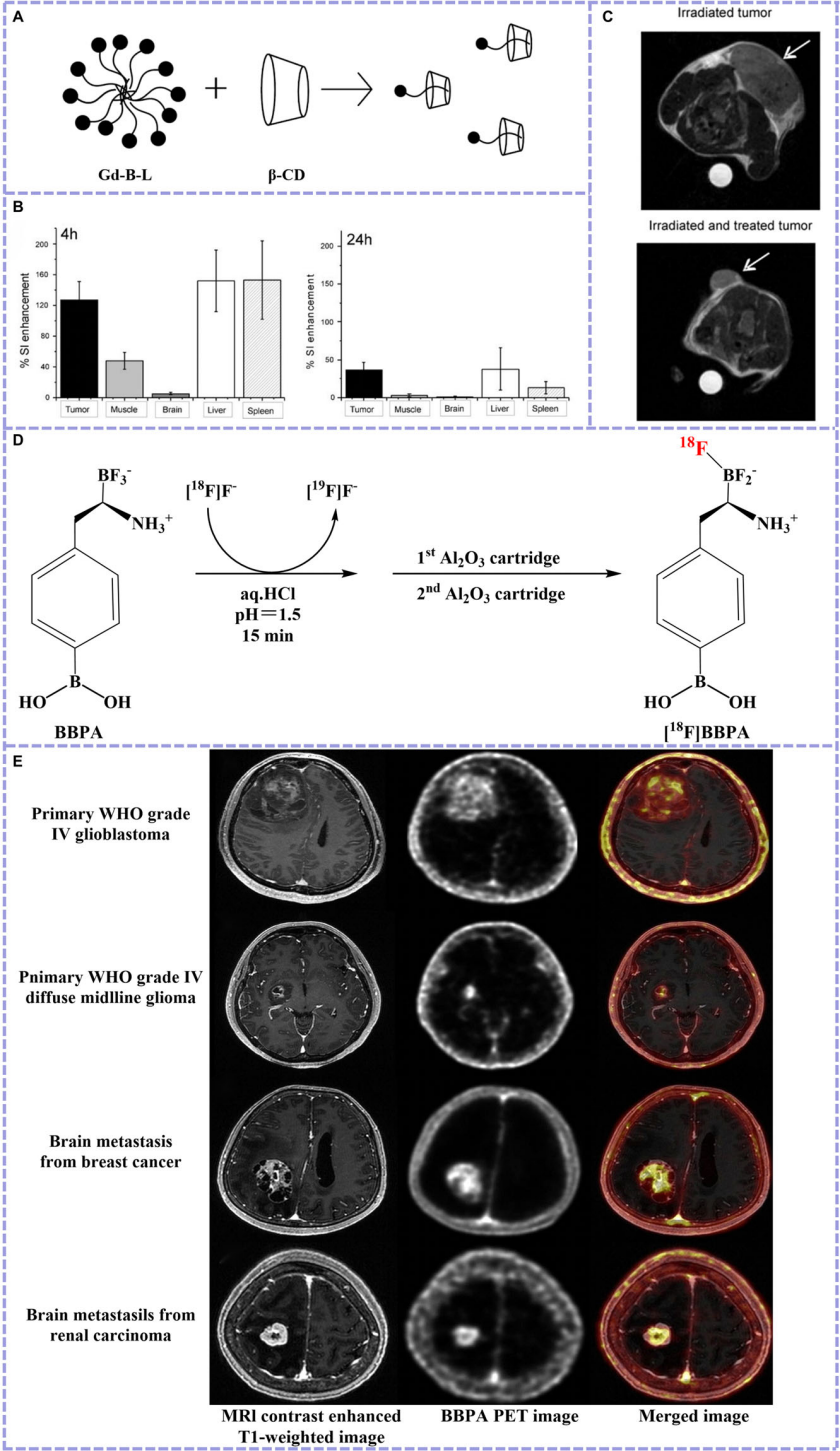
Preparation and evaluation of LDL particles loaded with Gd/B/L. (A) Schematic representation of the Gd/B/L–b‐CD supramolecular adduct formation. (B) A plot of MRI SI enhancements (%) measured in different organs 4 h and 24 h after the administration of the Gd/B/L–LDL adduct. (C) T_2_‐weighted Rapid Acquisition with Refocused Echoes (RARE) images acquired on tumor‐bearing mice irradiated with neutrons without (left) or after (right) administration of B atoms. The images were acquired 12 days after neutron irradiation. Reprinted with permission from Ref.^213^. Copyright 2011, ChemistryEurope. Preparation of [^18^F]BBPA and Examples of MRI and BBPA‐PET in Malignant Brain Tumors. (D) Preparation of [^18^F]BBPA. (E) Examples of MRI and BBPA‐PET in malignant brain tumors. (a) A primary glioblastoma (WHO grade IV, IDH wild‐type) with both contrast‐enhanced and non‐contrast‐enhanced components exhibited significant BBPA uptake, with SUVmax of 2.68 and T/N ratio of 18.4. (b) A primary diffuse midline glioma (WHO grade IV, H3K27M mutant) with moderate MRI contrast enhancement displayed elevated BBPA activity with SUVmax of 2.34 and a T/N ratio of 19.5. (c) A pathologically confirmed brain metastasis from breast cancer demonstrated significant BBPA uptake with SUVmax of 3.11 and T/N ratio of 26.9. (d) A pathologically confirmed brain metastasis from renal carcinoma exhibited significant BBPA activity with an SUVmax of 1.97 and a T/N ratio of 15.2. Elevated BBPA uptake can be observed in multiple neoplastic circumstances and is independent of MRI contrast enhancement pattern or extent. Reprinted with permission from Ref.^226^, ^227^. Copyright 2024, European Journal of Nuclear Medicine and Molecular Imaging.

Boron nuclide probes are specialized molecular tools that use boron elements to visualize specific structures within biological samples. These sophisticated probes are particularly suited for non‐optical high‐resolution imaging techniques such as Electron Energy Loss Spectroscopy and Secondary Ion Mass Spectrometry. By offering enhanced sensitivity and spatial resolution for imaging intracellular proteins, these probes facilitate diverse applications spanning direct target binding, immunostaining techniques, and metabolite analysis.^213^ Recent groundbreaking developments have significantly expanded the potential of boron‐based molecular probes in cancer diagnostics and treatment. In 2011, Geninatti‐Crich et al. pioneered a multimodal probe incorporating gadolinium as an MRI contrast agent, boron, and a ligand targeting low‐density lipoprotein (LDL) (Figure 9A).^214^ This innovative approach enabled quantitative tumor region monitoring in murine models, demonstrating significant tumor growth inhibition following neutron irradiation (Figure 9B, C).

Building on this foundation, Mishiro's research in 2022 introduced a multifunctional molecular probe combining boron clusters with tumor‐targeting peptides and radionuclide.^215^ By utilizing the fission properties of the radionuclide ^10^B and nuclear medicine imaging techniques, this approach enabled early lesion diagnosis and personalized and precise tumor treatment.

Additionally, fluorinated small‐molecule drugs have significantly impacted the pharmaceutical market.^216^, ^217^ In 2019, the FDA approved 13 new fluorinated drugs, accounting for 41% of all small‐molecule drugs.^218^ Given the status of fluorine as the second smallest atom after hydrogen, its high electronegativity, ability to form hydrogen bonds, influence on bond strength, and role as a conformational modulator, fluorination is an effective strategy for fine‐tuning final drug candidates to advance the drug discovery process.^219^, ^220^

However, fluorine substituents can act as bioisosteres, such as the CF3 group mimicking a C = O oxygen moiety^220^. On the other hand, fluorinated compounds can serve as diagnostic agents for PET applications.^221^, ^222^, ^223^ [^18^F]FDG, one of the most effective PET imaging agents for cancer diagnosis, has established the gold standard for detecting various tumors, including lung, head and neck, brain, and pancreatic cancers.^224^, ^225^

Most recently, Liu Zhibo's team designed [^18^F]BBPA, a novel broad‐spectrum cancer probe derived from trifluoroborates (Figure 9D). This probe demonstrated remarkable tumor uptake in B16‐F10 xenografts, significantly improving the tumor‐to‐brain ratio compared with conventional amino acid PET tracers (Figure 9E).^226^, ^227^ Notably, the uptake mechanism of [^18^F]BBPA functions independently of MRI contrast enhancement, offering a critical diagnostic alternative for challenging imaging scenarios where conventional methods prove inadequate.^227^

These advances represent significant progress in developing personalized cancer diagnostic and treatment strategies, underscoring the potential of boron‐based molecular probes in precision oncology.

## 4 CONCLUSION AND OUTLOOK

The evolution of BNCT technologies demonstrates a promising trajectory toward more effective, targeted approaches in cancer management, potentially transforming clinical outcomes for patients with diverse malignancies despite persistent clinical challenges. As an advanced, binary therapeutic approach, BNCT offers unique advantages, including precise targeting, enhanced safety, and potentially lower treatment costs compared with conventional cancer therapies. The fundamental mechanism of BNCT relies on the nuclear fission reaction of boron neutron capture, with L‐p‐boronophenylalanine (^10^BPA) currently standing as the sole FDA‐approved clinical drug. However, the clinical translation of BNCT remains hindered by critical limitations, primarily the insufficient tumor uptake of ^10^B and suboptimal T/N and T/B ratios.

Driven by advances in accelerator‐based neutron sources, innovative boron agent development, and increasingly sophisticated treatment planning systems, BNCT has entered a renewed phase of technological and translational progress with expanding biomedical relevance and clinical applicability. In particular, BNCT demonstrates considerable potential for the management of refractory and heterogeneous malignancies that remain difficult to treat with existing modalities. These include deep‐seated or inoperable tumors such as pancreatic cancer, non–small cell lung cancer, and brain metastases arising from breast or renal carcinomas, as well as hypoxic and treatment‐resistant cancers exemplified by glioblastoma multiforme, in which oxygen‐independent high–LET radiation may effectively circumvent established resistance mechanisms. Beyond primary tumor control, the inherent spatial selectivity of BNCT supports its potential role in eliminating minimal residual disease, an important yet unmet clinical need in preventing tumor recurrence. Furthermore, the capacity of BNCT to induce immunogenic cell death (ICD) through highly localized tumor cell destruction provides a mechanistic basis for rational combination strategies with immunotherapies or molecularly targeted agents, thereby positioning BNCT as a flexible and integrative therapeutic platform for addressing complex oncologic challenges.

The short tissue penetration range of radiation generated by ^4^He and ^7^Li underscores the paramount importance of precise subcellular targeting. Critically, the localization of ^10^B within specific tumor cellular compartments can dramatically amplify therapeutic efficacy due to increased absorbed dose. Moreover, targeting subcellular structures such as the nucleus and mitochondria can potentially increase biological effects by an order of magnitude compared to cytoplasmic localization. This refined targeting approach holds considerable clinical promise, offering the potential to reduce boron drug dosages while simultaneously enhancing the effectiveness of BNCT against tumors.

Advancing BNCT requires overcoming significant clinical challenges, particularly in real‐time, dynamic quantitative tracking of high‐abundance ^10^B concentrations and identifying patients with optimal ^10^B uptake potential. The precise timing of BNCT intervention emerges as a critical determinant of therapeutic success. Molecular imaging technologies present a transformative solution, enabling real‐time, quantitative visualization of ^10^B concentrations. This technological approach directly addresses two fundamental clinical imperatives: optimizing treatment regimens and stratifying patient populations most likely to benefit from the therapy. Future research initiatives should prioritize a comprehensive drug development strategy, focusing on the design and synthesis of multi‐targeted, multi‐polymeric radioactive ^10^B compounds. Key research objectives include enhancing tumor cell ^10^B uptake, extending ^10^B drug retention time, prolonging blood half‐life, and developing robust in vivo quantitative tracking methodologies. Achieving stringent clinical parameters, such as a tumor ^10^B uptake concentration of over 30 µg/g and T/N ratios of greater than 5, remains essential for BNCT therapeutic optimization.

The future trajectory of BNCT presents both promising opportunities and complex challenges. Emerging advances in molecular biology coupled with deepening understanding of tumor pathogenesis herald transformative potential for this therapeutic approach. Strategic approaches include developing novel boron delivery agents, designing innovative boron nuclide probes, and exploring synergistic treatment combinations. Particularly noteworthy is the potential combination therapy of BNCT and immune checkpoint inhibitors may create synergistic effects and increase lethality against tumor cells. The proposition that BNCT may facilitate the conversion of immunologically cold tumors into immunologically active ones has attracted increasing interest, as BNCT‐induced ICD can promote the release of tumor‐associated antigens and damage‐associated molecular patterns, thereby initiating antitumor immune responses. Nevertheless, this concept should currently be regarded as a plausible mechanistic hypothesis rather than an established therapeutic paradigm, given the biological complexity of tumor–immune interactions and the potential for unintended effects. In particular, the heterogeneous tumor microenvironment, characterized by the presence of immunosuppressive cell populations such as regulatory T cells and myeloid‐derived suppressor cells, together with inhibitory cytokines including transforming growth factor beta and interleukin 10, may substantially dampen immune activation triggered by ICD, thereby limiting effective immune reprogramming. Moreover, the combination of high‐LET radiation with immune activation raises the possibility of synergistic toxicities, including excessive inflammatory responses that could result in normal tissue injury or immune‐related adverse events. Accordingly, further systematic preclinical investigations and well‐designed clinical studies are required to define the conditions under which BNCT‐mediated immunomodulation may be most effective and safe. Key considerations include the selection of boron agents capable of enhancing ICD, optimization of the temporal relationship between neutron irradiation and immune status, and identification of patient subpopulations most likely to derive benefit. A rigorous understanding of these parameters will be essential to realize the immunotherapeutic potential of BNCT while minimizing associated risks. International collaborative efforts and robust interdisciplinary research will be instrumental in driving the evolution of BNCT. By integrating expertise from nuclear physics, chemistry, biology, and clinical medicine, researchers can unlock unprecedented therapeutic capabilities. The development of BNCT exemplifies the impact of collaborative, boundary‐crossing scientific innovation, bringing together advanced technologies and deep biological understanding with the aim of transforming cancer therapy.

The future success of BNCT depends not only on scientific and technological advancements but also on robust policy and financial support to facilitate smooth clinical research and equipment development. Equally critical is the cultivation of public and patient awareness, which will be instrumental in improving acceptance and driving broader clinical adoption of this emerging therapeutic modality. Despite being in its early developmental stages, continuous research and technological innovations hold significant promise, positioning BNCT as a potentially transformative tool in the cancer treatment arsenal. This innovative approach offers renewed hope to patients confronting this challenging disease, particularly those with limited treatment options or resistance to conventional therapies.

## CONFLICT OF INTEREST STATEMENT

The authors declare that they have no known competing financial interests or personal relationships that could influence the work reported in this paper.
